# Epidemiological analysis and risk factors of recurrent pulmonary tuberculosis in Western Zhejiang Province, China (2007–2024)

**DOI:** 10.3389/fpubh.2026.1777279

**Published:** 2026-03-04

**Authors:** Yide Hu, Min Wang, Yu Gao, Xiaogang Hao, Wei Wang, Kui Liu, Bingdong Zhan

**Affiliations:** 1 School of Public Health, Zhejiang Chinese Medicine University, Hangzhou, Zhejiang, China; 2 Quzhou Centre for Disease Control and Prevention, Quzhou, Zhejiang, China; 3 Zhejiang Provincial Center for Disease Control and Prevention, Hangzhou, China

**Keywords:** older adults, prevention, pulmonary tuberculosis, recurrence, risk factors

## Abstract

**Background:**

Recurrent pulmonary tuberculosis (PTB) after successful treatment is a significant challenge for current TB control efforts. This study investigated the epidemiological characteristics of recurrent PTB in western Zhejiang Province, aiming to identify risk factors for recurrence and predictors of retreatment outcomes, thereby providing a scientific basis for targeted prevention and management.

**Methods:**

This retrospective cohort study utilized surveillance data from the Tuberculosis Information Management System in Quzhou, Zhejiang, from January 1, 2007, to December 31, 2024. Recurrence-free intervals were analyzed to identify temporal patterns, and multivariable Cox proportional hazards regression models were employed to determine independent risk factors for recurrence and adverse retreatment outcomes.

**Results:**

Among 28,458 patients, 897 (3.15%) experienced recurrence. These cases were predominantly male (79.2%), older (mean age, 61.6 years), and farmers (80.3%). Recurrence-free intervals significantly shortened with subsequent episodes, dropping from a median of 804 days (95% CI, 740–900) for the first recurrence to 495 days (95% CI, 394–737) for the second (*p* < 0.001). Nearly half (48.9%) of all recurrences occurred within the first 2 years after treatment. Independent predictors of recurrence included advanced age (Adjusted HR, 1.77 for ≥65 years [95% CI, 1.46–2.16]), male sex (AHR, 1.42 [95% CI, 1.21–1.67]), and farming occupation (AHR, 1.22 [95% CI, 1.02–1.46]). For retreatment outcomes, age ≥65 years was the independent risk factor for adverse events (AHR, 1.91 [95% CI, 1.07–3.40]).

**Conclusion:**

Nearly half of the recurrences occurred within 2 years after treatment completion, and recurrence-free intervals progressively shortened with repeated episodes. The older population (≥65 years) faced a dual burden, identified as the primary risk group for both recurrence and adverse retreatment outcomes. For the risk factors of recurrence and the success of treatment, it is necessary to further refine and explore the influencing factors.

## 1 Introduction

Tuberculosis (TB) is a chronic infectious disease caused by *Mycobacterium tuberculosis* (MTB), which is mainly transmitted through the respiratory tract (1, 2) and is the leading cause of infectious disease deaths worldwide (3). The Global Tuberculosis Report 2025 indicates that 10.7 million new TB cases occurred globally in 2024 (incidence rate of 131 per 100,000), of which 15.89% were retreatment TB (4). Patients with retreatment TB included those who failed initial treatment and others who had recurrent TB after previous successful treatment (5). For the recurrent TB cases, evidence has found two sources of recurrence involved endogenous reactivated TB and exogenous reinfected TB (6). In low-incidence settings, the risk of reinfection is usually considered to be minimal, and most of the recurrent TB cases are expected to be due to reactivation (7). In contrast, in high-incidence areas, the proportion of recurrent TB cases due to reinfection is higher due to increased risk of exposure (8). Available literature demonstrated that patients with recurrent TB commonly require an additional round of treatment and require longer treatment duration, leading to reduced probability of treatment success and further contributing to additional transmission of MTB strains and increasing the burden of TB (9). This may present a challenge to END TB planning and the attainment of treatment objectives (10).

Several factors such as poor medication efficacy, poor adherence to anti-tuberculosis drugs, and the patient’s immune status (co-infection with HIV or diabetes mellitus), were related to the development and treatment outcome of recurrent TB (11–13). In addition, patients with recurrent TB have poorer treatment outcomes with high mortality, low treatment completion rates, and high rates of drug resistance compared to new-onset TB cases (14). Despite significant progress in TB control in China, however, limited efforts have been made to identify the main causes of recurrent TB in the country, including in Zhejiang Province. However, Quzhou City in the west of Zhejiang Province had accounted for the top case findings of all identifications, which had its potential value to identify the influencing factors of recurrent development (15). To help address these issues, we analyzed surveillance data to characterize the epidemiology of recurrent TB and identify specific demographic and clinical risk factors for both recurrence and adverse retreatment outcomes, which would contribute to the further development of targeted interventions for TB control.

## 2 Methods

### 2.1 Study area

**Figure 1:**
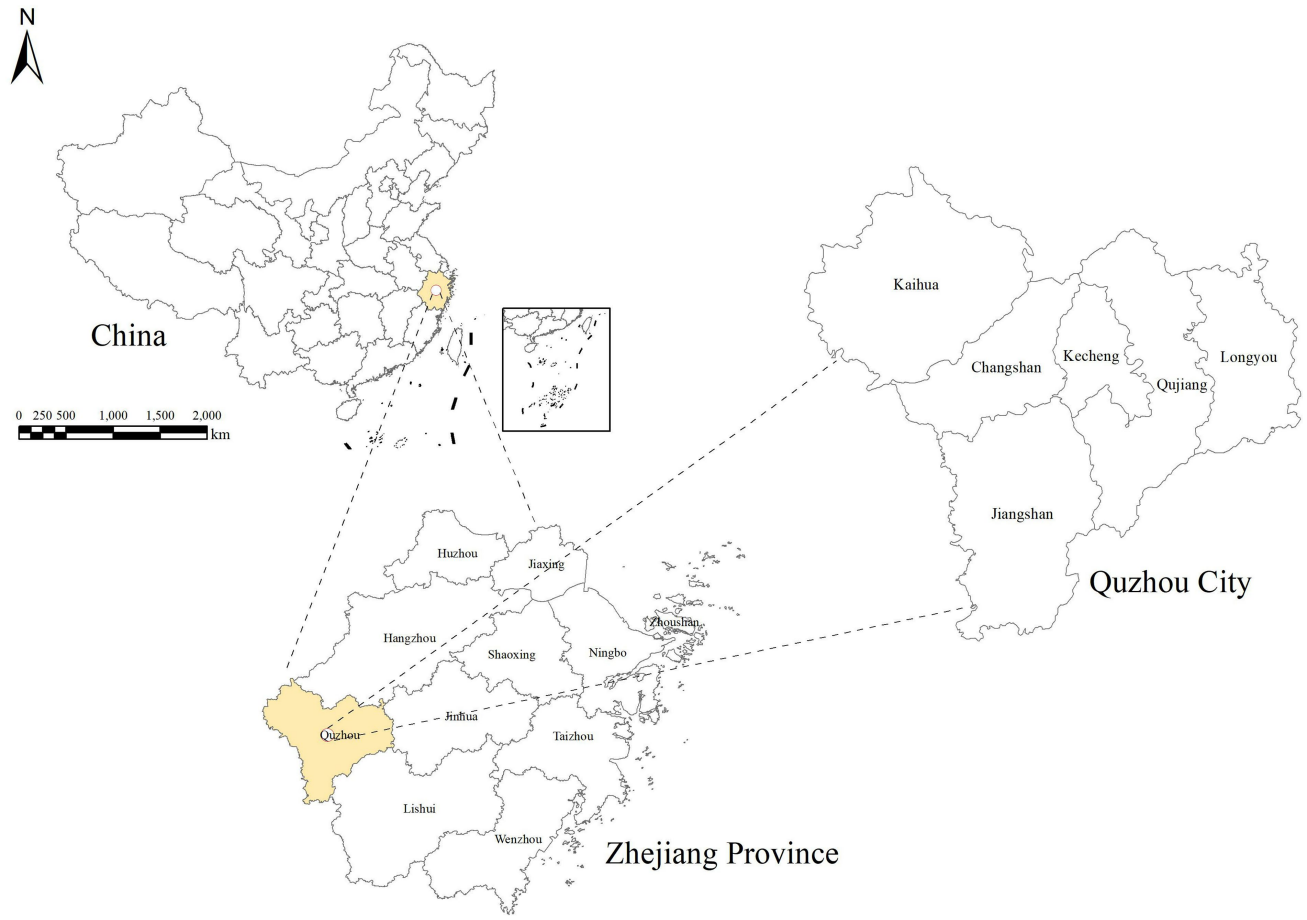
The location of Quzhou City. This study was conducted in Quzhou City, Zhejiang Province, China. The figure on the right shows the administrative divisions of Quzhou City, including Kecheng District, Qujiang District, Jiangshan City, Changshan County, Kaihua County, and Longyou County.

Quzhou City is situated in the western region of Zhejiang Province, China, covering an area of nearly 8,844 square kilometers, which represents 8.38% of the total area of Zhejiang Province. The city falls within the subtropical monsoon climate zone, characterized by four distinct seasons, namely “early spring and short autumn, long summer and winter.” It also experiences moderate temperatures, abundant sunlight, and noticeable occurrences of droughts and floods. Quzhou City encompasses six districts: Kecheng, Qujiang, Jiangshan, Longyou, Changshan, and Kaihua, with a resident population of 2,550,300 people and a GDP of 200.3 billion RMB in 2022. The geographical location of Quzhou City can be seen in Figure 1.

### 2.2 Study design and data collection

**Figure 2:**
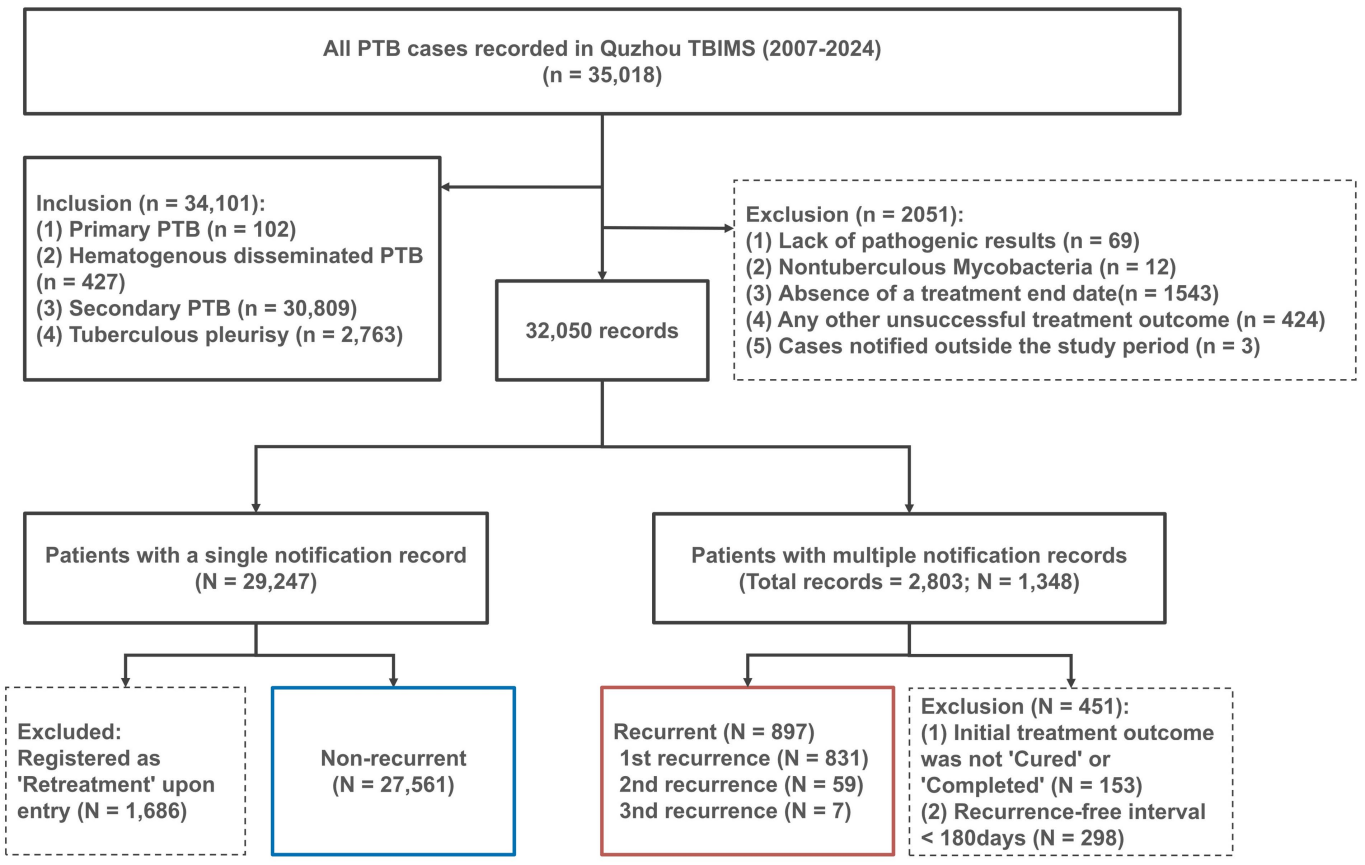
Flow chart for inclusion of patients with recurrent PTB in the study. Between 2007 and 2024, Quzhou Tubculosis Information Management System initially recorded a total of 35,018 tuberculosis cases. Through screening, a total of 28,458 patients were included in the final analysis. The chart describes in detail the inclusion and exclusion criteria of each stage of application. These included 27,561 newly diagnosed patients with only one report and 897 patients with recurrent tuberculosis screened, and dotted boxes indicate exclusion criteria. The strict definition of recurrence needs to meet two conditions: (1) The initial treatment result is cured or completed; (2) The interval between the completion of treatment and the second diagnosis is at least 180 days. Patients who do not meet these criteria are excluded. The final recurrence population continues to be classified according to (first, second, and third recurrence).

This study employed a retrospective cohort design utilizing longitudinal surveillance data. All the tuberculosis case data used in this study come from the tuberculosis information management system, which is extracted and analyzed by the Quzhou Center for Disease Prevention and Control under authorization. We collected data of PTB cases notified from January 1, 2007, to December 31, 2024, including population characteristics (sex, age, occupation, current residence, registered residence), clinical diagnosis information (centre of first contact, patient discovery method, and results of diagnosis). The inclusion criteria for the research subjects are as follows: (1) pulmonary lesions categorized as primary, hematogenous disseminated, and secondary PTB; (2) newly diagnosed TB for the first treatment episode; (3) classified as being cured or completing treatment as outcomes for their first treatment episode, in accordance with the guidelines of the national TB control plan; and (4) patients with the same or similar names (including phonetic similarities) and identical ID card numbers, or with the same names and genders and at least one matching item in age, registered residence address, current address, contact phone, or other information. The exclusion criteria for the research subjects are as follows: (1) lack of pathogenic results; (2) no pulmonary lesions (categorized as any kind of extra pulmonary tuberculosis alone) or nontuberculous mycobacteriosis (NTM); (3) missing a treatment end date; (4) an interval of less than 180 days between the completion of the previous treatment and the subsequent clinical diagnosis of PTB; and (5) any other unsuccessful treatment outcomes for the first treatment episode. Additionally, the demographic data for Quzhou City over the years were sourced from the “Quzhou Statistical Yearbook”. The details for the data collection were shown in Figure 2.

### 2.3 Definition

Recurrent PTB refers to individuals who have a documented history of TB, have been deemed cured or completed the prescribed treatment by a physician, but have subsequently been re-diagnosed with TB after remaining TB-free for at least 6 months following the completion of the most recent anti-tuberculosis treatment (16).

According to the Chinese Technical Code for Tuberculosis Prevention and Control (2020 Edition) (17), successful treatment includes both cure and completion of the course. Cure is achieved when bacteriologically positive patients complete the prescribed treatment, with negative sputum smears or cultures at the end of the last month of the course and at the final smear or culture test. Completion of the course is defined as the fulfillment of the prescribed treatment for pathogen-negative patients, with negative or no sputum test results on smears or cultures at the end of the treatment, and for pathogen-positive patients, with no sputum test results at the end of the treatment but with negative sputum results on the most recent smears or cultures. The date of TB recurrence was strictly defined as the date of clinical confirmation. Case finding for tuberculosis primarily relies on symptomatic visits and active screening. Adverse treatment outcomes encompass treatment failure, adverse drug reactions, loss to follow-up, death (regardless of cause during treatment), and conversion to multidrug-resistant therapy. Urban areas were non-agricultural residential zones with concentrated populations, typically found in towns at or above the county level; rural areas were defined as locations outside these urban areas. The resident population who had lived in Quzhou for more than 6 months, while the migrant population consisted of individuals who, for various reasons, relocated to Quzhou City from their registered residence less than 6 months.

### 2.4 Statistical analysis

This study adopts a descriptive analysis method, including recurrence rate and composition ratio, to describe the general demographic characteristics of recurrence in PTB patients. The difference between recurrent PTB and non-recurrent PTB in the baseline is compared with the chi-square test; for variables that do not meet the chi-square test conditions, the Fisher’s exact test was used. Annual notification rates were evaluated using quasi-Poisson regression to account for overdispersion. The median recurrence-free interval was calculated, and its 95% confidence interval (CI) was estimated using bootstrap resampling; recurrence-free intervals across recurrence orders were compared using the Kruskal-Wallis test and the Wilcoxon rank-sum test. Cumulative recurrence was estimated using the Kaplan–Meier method, and subgroup differences were assessed with the log-rank test. Cox proportional hazards regression was used to examine factors associated with TB recurrence and with unfavorable treatment outcomes. For the time-to-event analysis of treatment outcomes, patients with invalid or missing data regarding treatment duration (n = 6, 0.7%) were excluded from the model to ensure estimation accuracy. Consequently, the final multivariate analysis for adverse outcomes was based on a cohort of 891 patients. The proportional hazards assumption was verified using Schoenfeld residuals and visual inspection of the log-minus-log survival plots. No substantial violations were observed. Variables with *p* < 0.05 in univariable analyses were entered into multivariable models, and backward stepwise selection was used to derive the final model. To assess temporal robustness, sensitivity analyses were conducted by adjusting for study period (2007–2016 and 2017–2024) and stratifying by these eras to examine potential effect modification. Results were reported as hazard ratios (HRs) with 95% CIs. All analyses were conducted in R (version 4.5.1), and a 2-sided *p* < 0.05 was considered statistically significant.

## 3 Results

### 3.1 Baseline characteristics of patients with recurrent PTB

During the study period, a total of 897 recurrent PTB cases were notified in Quzhou City, representing 3.15% of all PTB cases (*n* = 28,458). More than two-thirds (79.2%) of the individuals diagnosed with recurrent PTB were male (*n* = 710). The average age of these patients was 61.6 years old, of which patients aged 65 and above constitute the largest group (*n* = 400, 44.6%). Among the cases of recurrent PTB, most of them live in rural areas (*n* = 648, 72.2%), of which 80.3% (*n* = 720) are farmers, and the resident population accounts for 91.8% (*n* = 823). More than half (*n* = 467, 52.1%) of the patients were initially diagnosed at municipal medical institutions. Recurrent PTB is mainly diagnosed through symptomatic visits (*n* = 492, 54.8%). Regarding diagnostic results, 48.8% (*n* = 438) of the cases were positive for bacteriological testing, while 51.2% (*n* = 459) of the cases were negative for bacteriological testing. The baseline characteristics of patients with recurrent PTB are detailed in Table 1.

**Table 1:** Baseline characteristics of patients with recurrent and non-recurrent tuberculosis.

Characteristics	Total (*N* = 28,458)	Non-recurrent PTB (*N* = 27,561)	Recurrent PTB (*N* = 897)	*p*-value
Sex, n (%)				<0.001
Female	8,074 (28.4%)	7,887 (28.6%)	187 (20.8%)	
Male	20,384 (71.6%)	19,674 (71.4%)	710 (79.2%)	
Ethnicity, n (%)				0.23
Han	28,256 (99.3%)	27,362 (99.3%)	894 (99.7%)	
Non-Han	202 (0.7%)	199 (0.7%)	3 (0.3%)	
Age, n (%)				<0.001
0–44 years old	8,499 (29.9%)	8,338 (30.3%)	161 (17.9%)	
45–64 years old	9,107 (32.0%)	8,771 (31.8%)	336 (37.5%)	
> = 65 years old	10,852 (38.1%)	10,452 (37.9%)	400 (44.6%)	
Occupation, n (%)				<0.001
Non-farmer	8,450 (29.7%)	8,273 (30.0%)	177 (19.7%)	
Farmer	20,008 (70.3%)	19,288 (70.0%)	720 (80.3%)	
Current residence, n (%)				<0.001
Urban	9,935 (34.9%)	9,686 (35.1%)	249 (27.8%)	
Rural	18,523 (65.1%)	17,875 (64.9%)	648 (72.2%)	
Notification, n (%)				<0.001
Resident population	25,377 (89.2%)	24,554 (89.1%)	823 (91.8%)	
Migrant population	3,081 (10.8%)	3,007 (10.9%)	74 (8.2%)	
Centre of first contact, n (%)				<0.001
County medical institutions	11,058 (38.9%)	10,628 (38.6%)	430 (47.9%)	
Municipal medical institutions	17,400 (61.1%)	16,933 (61.4%)	467 (52.1%)	
Patient discovery method, n (%)				<0.001
Symptomatic visits	13,794 (48.5%)	13,302 (48.3%)	492 (54.8%)	
Active screening	2092 (7.4%)	2073 (7.5%)	19 (2.1%)	
Referral	10,439 (36.7%)	10,143 (36.8%)	296 (33.0%)	
Other	2,133 (7.5%)	2,043 (7.4%)	90 (10.0%)	
Diagnosis, n (%)				<0.001
Negative	16,739 (58.8%)	16,280 (59.1%)	459 (51.2%)	
Positive	11,719 (41.2%)	11,281 (40.9%)	438 (48.8%)	

### 3.2 Trends in recurrent PTB

**Figure 3:**
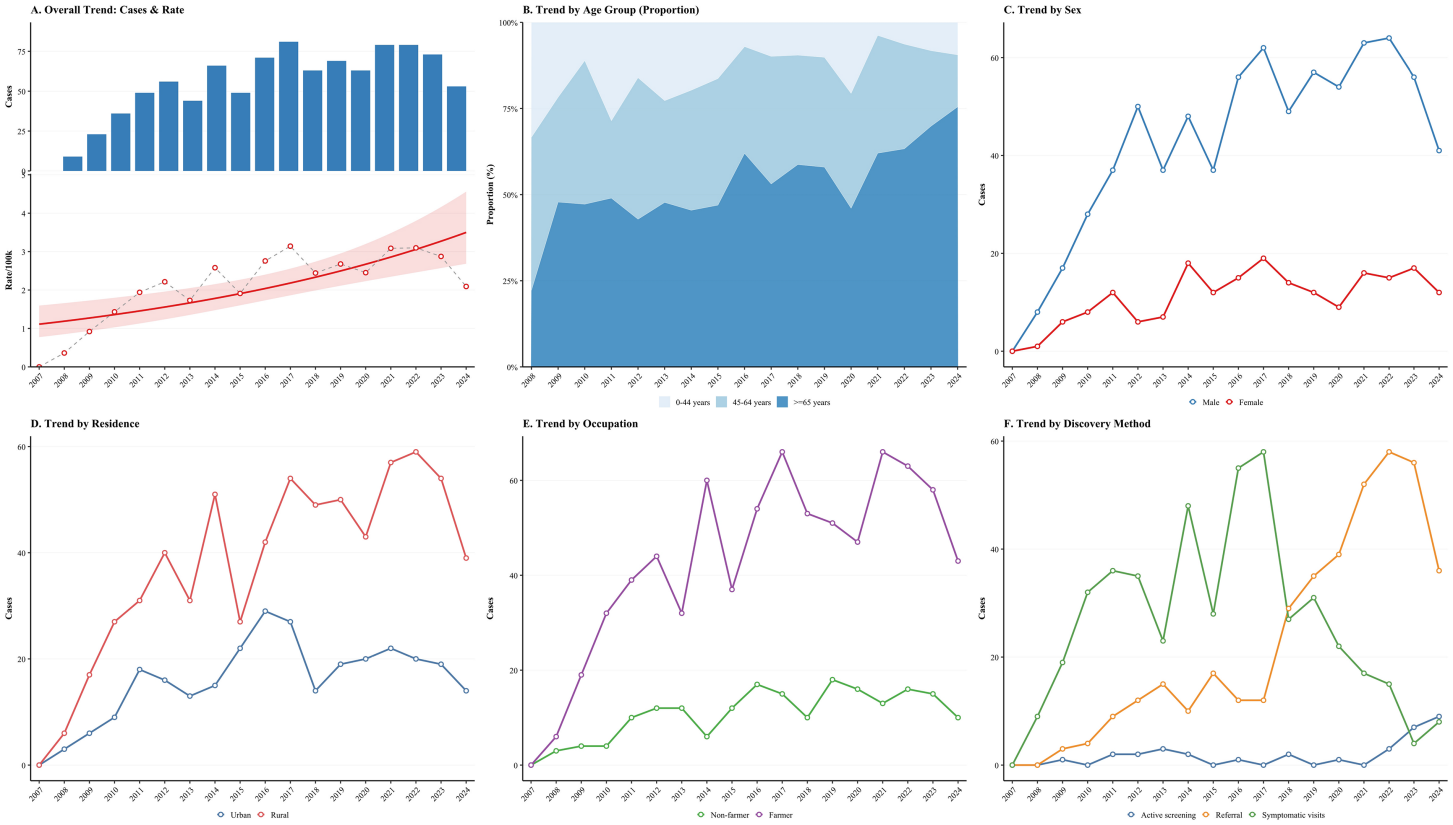
Temporal trends and demographic shifts in recurrent pulmonary tuberculosis in Quzhou, 2007–2024. As shown in the figure, we analyzed the longitudinal epidemiological characteristics of 897 cases of recurrent tuberculosis. **(A)** The trend of the overall burden of disease each year. The blue bar chart shows the absolute number of recurrent cases, and the red dot shows the incidence of the corresponding year (per 100,000 population). The red solid line is obtained by fitting the quasi-Poisson regression model (the shadow area indicates its 95% confidence interval). **(B)** The changes in the age structure, especially the increasing trend of the proportion of older adult patients (≥65 years old), and the total annual number of recurrent cases used as denominators for **(B)** are shown in **(A)**. **(C–E)** The line charts of the number of cases respectively, this is by gender (male vs. female), place of residence (rural vs. city), occupation (farmer vs. non-farmers) for classification. **(F)** The trend of case detection, distinguishing between passive detection (symptomatic visits, referral) and active screening.

The longitudinal analysis revealed that the epidemiological pattern of PTB cases has changed during the 18-year study (Figure 3). According to the quasi-Poisson regression model, the annual reporting rate of recurrent tuberculosis fluctuates, but there has been an overall downward trend since 2017 (Figure 3A). The demographic characteristics of recurrent cases tend to age, and the proportion of recurrent patients aged 65 and above is gradually increasing (Figure 3B). In addition, the absolute number of recurrent cases among men, rural residents and farmers has always been higher than their respective control groups (Figures 3C–E). Regarding case detection, although symptomatic consultation is still the main detection method, the contribution of active screening has changed over time (Figure 3F).

### 3.3 Recurrence dynamics and cumulative incidence

**Figure 4:**
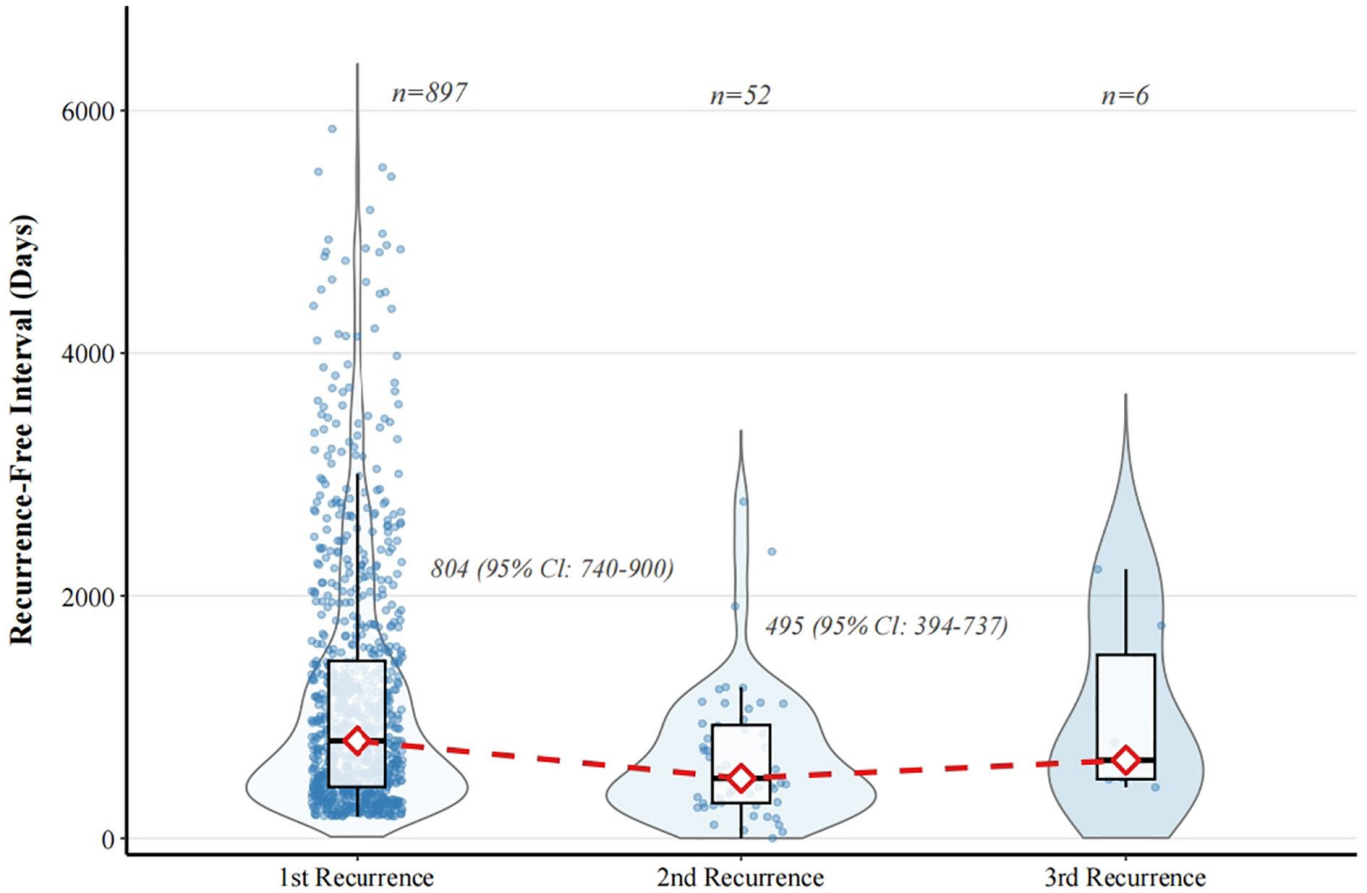
Changes in recurrence-free interval by episode frequency, 2007–2024. The figure shows the distribution of no recurrence interval (days) of patients with the first recurrence (*n* = 897), the second recurrence (*n* = 52), and the third recurrence (*n* = 6). The red diamond with the dotted line represents the median interval of no recurrence of each group. From the first recurrence (804 days; 95% CIl: 740–900 days) to the second recurrence (495 days; 95% CI: 394–737 days), the median interval was significantly shortened. And it has statistical significance (*p* < 0.001) after the Wilcoxon rank sum test. And the 95% confidence interval of the two did not coincide (using the Bootstrap method), indicating that the second recurrence interval of recurrent tuberculosis was significantly shortened and the recurrence of the disease accelerated. Note: Seven cases were excluded from the second recurrence analysis because the recorded date of recurrence preceded the completion date of the previous treatment, resulting in invalid (negative) intervals.

The analysis of recurrence-free interval reveals that there is a significant “acceleration phenomenon” in subsequent recurrence events (Figure 4). The median recurrence-free interval was significantly shortened, from 804 days (IQR, 350–1,456 days) of the first recurrence to 495 days (IQR, 269–906 days) of the second recurrence. Moreover, the 95% confidence intervals of 740–900 days and 394–737 days do not overlap with each other. Combined with the Wilcoxon rank sum test (*p* < 0.001), it is confirmed that the interval between the second recurrence is significantly shorter than the interval of the first recurrence. Due to the limited sample size, it is impossible to conduct a formal statistical comparison, so the data of the third recurrence (*n* = 6) is relatively biased.

**Figure 5:**
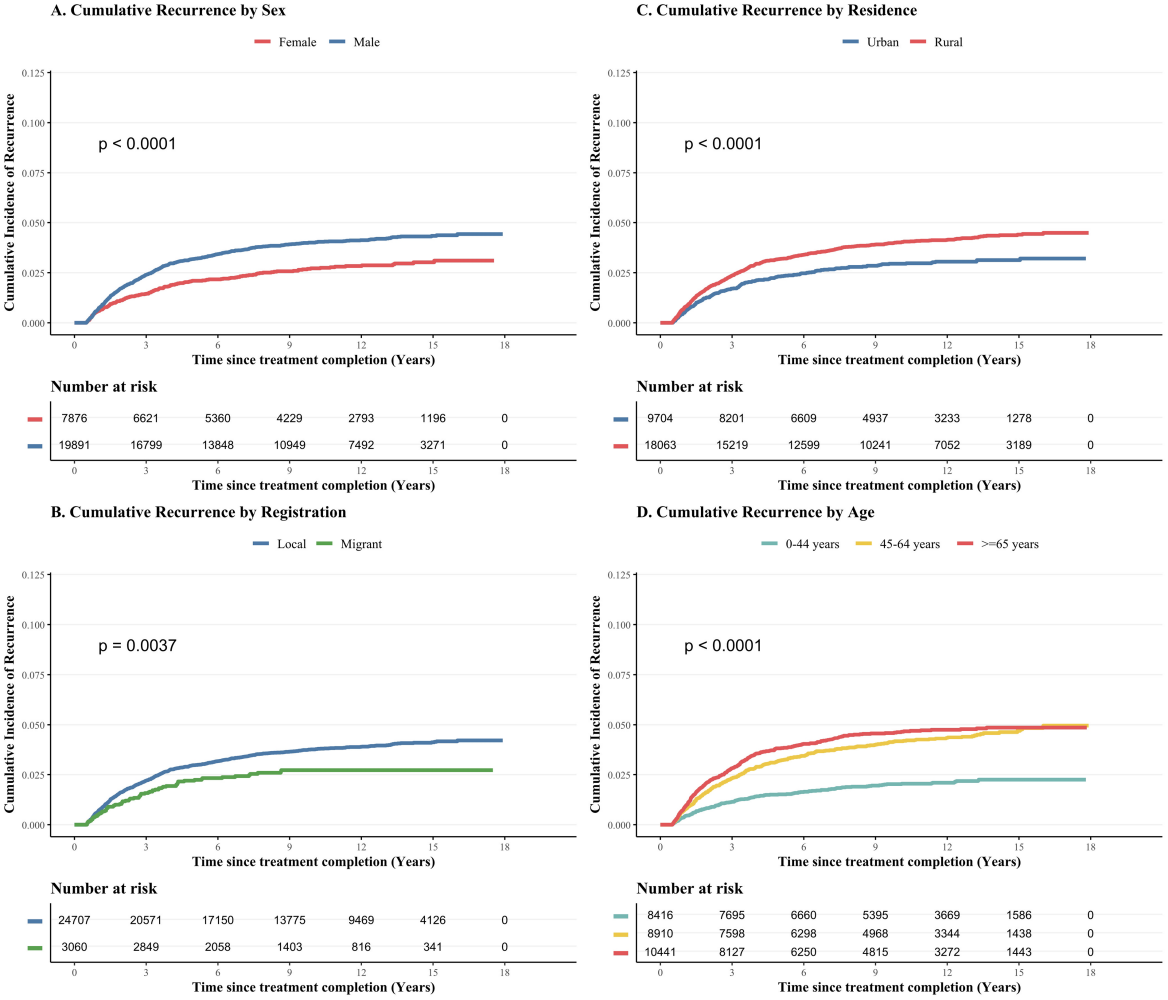
Cumulative incidence of tuberculosis recurrence according to key demographic characteristics, 2007–2024. The chart uses the Kaplan–Meier method to statistically analyze the cumulative recurrence rate of 28,458 patients who have completed the initial course of tuberculosis treatment. This study takes the time between the completion of the initial treatment and the first diagnosis of recurrence as the evaluation index. The chart shows the cumulative incidence by gender **(A)**, household registration type **(B)**, current residential area **(C)**, and age group at the time of first diagnosis **(D)**. The schedules in each chart list the statistics of the number of at-risk populations in a three-year observation cycle. Statistical significance is determined by the numerical rank test method.

Kaplan–Meier survival analysis shows that there are significant differences in the cumulative recurrence rate of different subgroups (Figure 5). The cumulative incidence of recurrence was significantly higher in males compared to females (log-rank *p* < 0.001; Figure 5A) and in rural residents compared to urban residents (log-rank *p* < 0.001; Figure 5C). In addition, the cumulative recurrence rate of older groups (45–64 years old and ≥65 years old) is significantly higher than that of the 0-44-years age group (log-rank *p* < 0.001; Figure 5D). A significant difference was also noted based on household registration, with local residents demonstrating higher recurrence rates than the migrant population (log-rank *p* = 0.004; Figure 5B).

### 3.4 The influencing factors of recurrent PTB using multivariate analysis

Multivariate analysis reveals the risk factors of tuberculosis recurrence. In the multivariate Cox regression analysis (Table 2), several independent risk factors associated with recurrence were identified after adjusting the potential concomplication factors. Advanced age was strongly associated with an increased risk of recurrence; compared to patients aged 0–44 years, the risk was significantly higher for those aged 45–64 years (Adjusted HR, 1.70 [95% Cl, 1.40–2.07]) and for those aged 65 years or older (AHR, 1.77 [95% CI, 1.46–2.16]). Male (AHR, 1.42 [95% CI, 1.21–1.67]) and positive bacterial (AHR, 1.38 [95% CI, 1.20–1.58]) are also important predictors of recurrence. Occupation as a farmer remained an independent risk factor (AHR, 1.22 [95% CI, 1.02–1.46]). Conversely, protective factors were also identified. Compared with patients treated in county level medical institutions, patients who received initial treatment in municipal-level medical institutions have a lower risk of recurrence (AHR, 0.82 [95% CI, 0.73–0.92]). In addition, in the patient discovery method, compared with symptomatic visits through active screening, the former is associated with a significant reduction in the risk of recurrence (AHR, 0.48 [95% CI, 0.30–0.77]). In the sensitivity analysis adjusting for the study period, the period variable itself was not statistically significant (*p* = 0.97), and the hazard ratios for core predictors remained virtually unchanged (Supplementary Table 3). Stratified analysis by period revealed that while male sex and advanced age were consistent risk factors, occupation as a farmer emerged as a more prominent predictor in the recent era (2017–2024) (Supplementary Table 2).

**Table 2:** Cox proportional hazards analysis of risk factors for tuberculosis recurrence.

Characteristics	Univariate analysis			Multivariable analysis	
	N	Crude HR (95% CI)	*P*-value	Adjusted HR (95% CI)	*P*-value
Sex					
Female	7,876	1.00 (Reference)			
Male	19,891	**1.49 (1.27–1.75)**	**<0.001**	**1.42 (1.21–1.67)**	**<0.001**
Age					
0–44 years old	8,416	1.00 (Reference)			
45–64 years old	8,910	**2.10 (1.74–2.53)**	**<0.001**	**1.70 (1.40–2.07)**	**<0.001**
≥65 years old	10,441	**2.32 (1.93–2.78)**	**<0.001**	**1.77 (1.46–2.16)**	**<0.001**
Occupation					
Non-farmer	8,260	1.00 (Reference)			
Farmer	19,507	**1.73 (1.47–2.04)**	**<0.001**	**1.22 (1.02–1.46)**	**0.027**
Current residence					
Urban	9,704	1.00 (Reference)			
Rural	18,063	**1.38 (1.20–1.60)**	**<0.001**		
Registration					
Resident population	24,707	1.00 (Reference)			
Migrant population	3,060	**0.70 (0.56–0.89)**	**0.004**		
Centre of first contact					
County medical institutions	10,866	1.00 (Reference)			
Municipal medical institutions	16,901	**0.73 (0.64–0.83)**	**<0.001**	**0.82 (0.73–0.92)**	**0.004**
Patient discovery method					
Symptomatic visits	13,743	1.00 (Reference)			
Active screening	1,907	**0.31 (0.20–0.50)**	**<0.001**	**0.48 (0.30–0.77)**	**0.002**
Referral	10,017	1.07 (0.93–1.24)	0.358	0.98 (0.84–1.13)	0.764
Other	2,100	**1.35 (1.08–1.69)**	**0.009**	1.16 (0.92–1.47)	0.217
Diagnosis					
Negative	16,561	1.00 (Reference)			
Positive	11,206	**1.66 (1.46–1.90)**	**<0.001**	**1.47 (1.29–1.68)**	**<0.001**
Study period					
Period 1 (2007–2016)	17,886	1.00 (Reference)			
Period 2 (2017–2024)	9,881	0.99 (0.84–1.16)	0.875		

CI, Confidence Interval; HR, Hazard Ratio; Backward stepwise selection was used. This analysis is based on a total of 28,458 research subjects (897 cases of recurrence and 27,561 cases of non-recurrence). Multivariate analysis: use the backward step-by-step selection method (based on the AIC criterion) to determine independent risk factors. Diagnosis: “Negative” includes cases with negative bacteriological examination and cases of tuberculosis pleurisy. Bold values indicate statistical significance (*p* < 0.05).

### 3.5 The influencing factors for the treatment outcome of recurrent PTB using multivariate analysis

**Figure 6:**
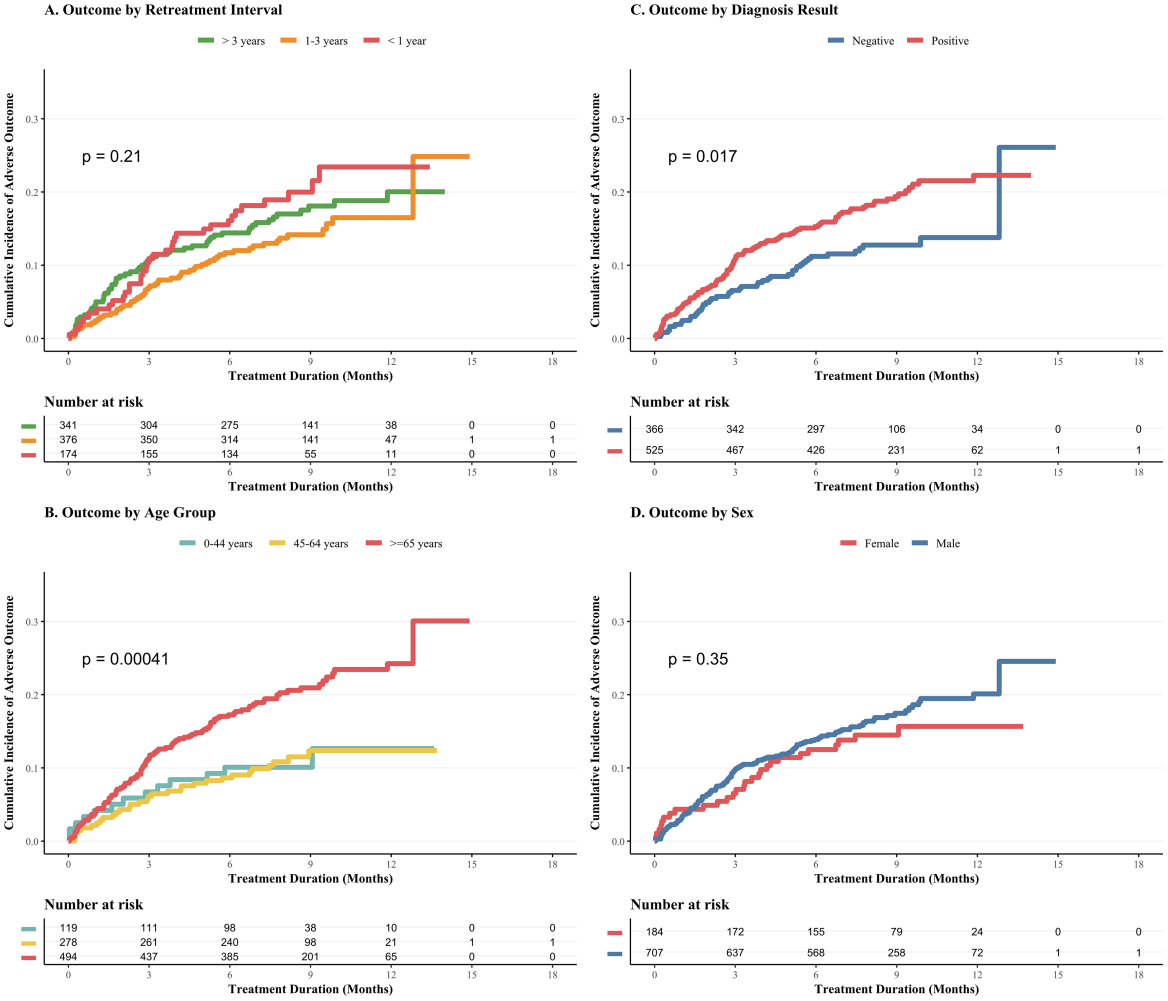
Cumulative incidence of adverse treatment outcomes during the first recurrence episode, 2007–2024. For the group with recurrence of tuberculosis, we evaluated the cumulative occurrence of adverse clinical transformation. Adverse conversion covers ineffective treatment, death, loss of contact, and other negative outcomes, while effective treatment (including recovery or completion of treatment) is treated as truncated data. **(A)** The cumulative incidence statistics based on the no-recurrence interval (that is, the time span from the initial cure to the recurrence confirmation). No significant statistical differences were found between the three groups (*p* = 0.21). **(B–D)** The cumulative incidence distribution according to different age groups, bacteriological diagnosis results, and gender. Especially in **(B)**, it was found that there was significant statistical significance between the three groups (*p* < 0.001). All statistical significance differences are evaluated by the numerical rank test method.

Among the 891 patients with recurrent PTB who underwent retreatment, 740 (83.1%) achieved treatment success, defined as either cure or completion, while 151 (16.9%) experienced adverse outcomes. Kaplan–Meier analysis of adverse outcomes (Figure 6) shows that no significant correlation between recurrence intervals (*p* = 0.21) (Figure 6A) and gender (*p* = 0.35) (Figure 6D) and adverse outcomes were not found. However, the cumulative incidence of adverse outcomes was significantly higher in patients aged 65 years or older (*p* < 0.001) (Figure 6B). In the univariate Cox proportional regression analysis (Table 3), age ≥65 years old (HR, 2.00 [95% CI, 1.12–3.55]) and bacteriological positive diagnosis (HR, 1.52 [95% CI, 1.08–2.15]) were significantly related to adverse treatment outcomes. In the multivariate cox analysis, after adjusting the potential mixing factors, the age of ≥65 is still a significant independent risk factor (AHR, 1.91 [95%CI, 1.07–3.40]). The study period was not an independent predictor of adverse outcomes (*p* = 0.13) after adjustment (Supplementary Table 3). However, stratified analysis indicated that the risk of adverse outcomes associated with advanced age (≥65 years) was attenuated in the recent period compared to the earlier period, although the protective effect of active screening remained directionally consistent (Supplementary Tables 2, 3).

**Table 3:** Cox proportional-hazards analysis of risk factors for adverse treatment outcomes among patients with recurrent pulmonary tuberculosis.

Characteristics			Univariate analysis		Multivariable analysis	
	Successful treatment (*N* = 740)	Adverse outcomes (*N* = 151)	Crude HR (95% CI)	*P*-value	Adjusted HR (95% CI)	*P*-value
Sex, n (%)						
Female	157 (85.3%)	27 (14.7%)	1.00 (Reference)			
Male	583 (82.5%)	124 (17.5%)	1.22 (0.80–1.85)	0.355		
Age, n (%)						
0–44 years old	106 (89.1%)	13 (10.9%)	1.00 (Reference)			
45–64 years old	246 (88.5%)	32 (11.5%)	1.00 (0.52–1.90)	0.989	0.98 (0.51–1.88)	0.962
≥ 65 years old	388 (78.5%)	106 (21.5%)	**2.00 (1.12–3.55)**	**0.019**	**1.91 (1.07–3.40)**	**0.029**
Occupation, n (%)						
Non-farmer	151 (83.0%)	31 (17.0%)	1.00 (Reference)			
Farmer	589 (83.1%)	120 (16.9%)	0.98 (0.66–1.46)	0.934		
Current residence, n (%)						
Urban	228 (85.4%)	39 (14.6%)	1.00 (Reference)			
Rural	512 (82.1%)	112 (17.9%)	1.21 (0.84–1.75)	0.300		
Registration, n (%)						
Resident population	678 (82.9%)	140 (17.1%)	1.00 (Reference)			
Migrant population	62 (84.9%)	11 (15.1%)	0.90 (0.49–1.66)	0.730		
Centre of first contact, n (%)						
County medical institutions	334 (84.6%)	61 (15.4%)	1.00 (Reference)			
Municipal medical institutions	406 (81.9%)	90 (18.1%)	1.20 (0.87–1.66)	0.274		
Patient discovery method, n (%)						
Symptomatic visits	365 (83.1%)	74 (16.9%)	1.00 (Reference)			
Active screening	31 (93.9%)	2 (6.1%)	0.34 (0.08–1.40)	0.135		
Referral	293 (81.2%)	68 (18.8%)	1.10 (0.79–1.53)	0.584		
Other	51 (87.9%)	7 (12.1%)	0.69 (0.32–1.50)	0.351		
Diagnosis, n (%)						
Negative	319 (87.2%)	47 (12.8%)	1.00 (Reference)			
Positive	421 (80.2%)	104 (19.8%)	**1.52 (1.08–2.15)**	**0.017**	1.42 (1.00–2.01)	0.049
Study period, n (%)						
Period 1 (2007–2016)	64 (16.8%)	317 (83.2%)	1.00 (Reference)			
Period 2 (2017–2024)	87 (17.1%)	423 (83.3%)	0.96 (0.69 to 1.33)	0.816		

OR, odds ratio; CI, confidence interval. As shown in the table, this analysis is based on 891 cases of recurrent tuberculosis patients. Among the 897 confirmed cases of recurrence, 6 cases were excluded because the treatment duration data was invalid or missing (time ≤0). Adverse outcomes include treatment failure, death, missed visit or other adverse outcomes, while successful treatment (cure or completion of treatment) is considered truncated data. In multivariate analysis, the backward elimination method is used to select the variables to be included in the multivariate analysis. Bold values indicate statistical significance (*p* < 0.05).

## 4 Discussion

Although TB is recognized as a preventable and treatable disease, it still contributes to a considerable global burden of disease (16, 18, 19). Patients with recurrent PTB are usually more serious, face greater difficulties in medication adherence, poor health, and need to receive a second or even multiple treatment regimen, which may increase the risk of drug resistance of *Mycobacterium tuberculosis* (20, 21). In order to solve the problems of the high rate of recurrent PTB and unsatisfactory treatment results. Our study was the first to explore the epidemiological characteristics, changing trends, and risk factors of recurrent PTB in western Zhejiang. The results of the study showed that recurrence remains a challenge, although the majority of recurrent cases achieved treatment success. Among them, males were more likely to be recurrent than females, and older TB patients were more likely to be recurrent. Although the rate of recurrent PTB in Quzhou exhibited fluctuations, it has shown a downward trend since 2017. The results of this study can provide a reference for future policy formulation and research on the prevention and treatment of recurrent PTB in China.

Recurrent PTB is defined as a new TB episode in patients who have been considered cured after completing treatment (22). Its epidemiological characteristics vary significantly across different regions and countries, generally mirroring the local TB burden. In low-incidence settings—such as Israel, Finland, the United Kingdom, and the United States—recurrence rates are typically low, ranging from 0.6 to 1.8% (23–27). Conversely, recurrence rates are substantially elevated in high-burden regions or high-risk populations. For instance, a pooled analysis of Indian cohorts reported a recurrence rate as high as 8% (28), and a systematic review indicated that recurrence estimates could range broadly from 4.9 to 47% depending on regional epidemiology and study definitions (22, 29). In our study, the recurrent PTB rate was 3.15%. This figure places our region in an intermediate position globally. It is higher than the average recurrence rates observed in European countries (30, 31) and the 2.06% recurrence rate reported by Shao et al. in Jiangsu, China (32). However, it remains significantly lower than the rates reported in high-burden countries such as India (10.9%) (33) and South Africa (11%) (34). The variation in recurrence rates likely reflects differences in the baseline TB incidence, as well as variations in risk factors that drive both infection rates and the progression to active disease (35).

Regarding the temporal dynamics of recurrence, existing literature consistently identifies the initial post-treatment phase as the critical risk window, with the majority of recurrences occurring within 1–2 years (11, 26, 36, 37). For instance, data from South Korea indicated that 50.3% of recurrences occurred within the first year (37), while a Dutch study quantified the reactivation rate at 228/100,000 person-years in the first 2 years, dropping to 57 thereafter (37). In our study, we conducted a granular analysis of these intervals and observed a novel “acceleration phenomenon” in repeated episodes. Specifically, the median recurrence-free interval significantly shortened for the first recurrence compared to the second recurrence. This rapid progression in subsequent episodes suggests a cumulative compromise in host immunity or a persistent endogenous reservoir. This aligns with the biological distinction between endogenous reactivation and exogenous reinfection. In low-incidence settings, reactivation is the predominant driver, accounting for approximately 80% of recurrent cases (24, 25, 38). The acceleration we observed might indicate a cumulative compromise in host defense mechanisms, highlighting that patients with a history of recurrence are progressively more vulnerable and warrant intensified, prolonged surveillance beyond the standard regimen.

Recurrence is the result of a complex interplay involving host susceptibility, treatment-related factors, and environmental contexts. In our multivariable Cox regression analysis, male sex was identified as an independent risk factor for recurrence. This finding aligns with a broad consensus in the literature identifying male gender as a high-risk determinant (23, 28, 36), although it contrasts with a previous systematic review from India which found no such association (39). The male predisposition to recurrence may be attributable to behavioral factors, such as higher rates of smoking (11, 37), alcohol consumption (11, 22, 29), and generally poorer treatment adherence compared to females. Additionally, we observed that advanced age is a robust predictor of recurrence. Compared to patients aged 0–44 years, the risk was significantly elevated for those aged 45–64 years and ≥65 years. This is consistent with earlier studies highlighting the vulnerability of older populations (40, 41). We speculate that this association is driven by immunosenescence—the gradual decline in immune function with age—and the higher prevalence of comorbidities. Conditions such as diabetes mellitus (22, 36), chronic lung disease (25), and malnutrition (low BMI) (28), which are more common in the older adults, jeopardize the host’s ability to maintain containment of Mtuberculosis, thereby facilitating both the rekindling of primary occult foci and susceptibility to exogenous reinfection.

Socioeconomic status and accessibility to healthcare systems also play a crucial role. In China, a significant proportion of the TB burden is concentrated in rural areas (42), complicating disease control efforts (43). Our study confirms that farmer status is an independent risk factor for tuberculosis recurrence. These associations should be interpreted as surrogate indicators of broader socioeconomic and healthcare system determinants rather than independent biological risk factors. Rural populations often face multiple barriers to accessing healthcare, such as long distances to designated TB hospitals and lower health literacy, which may increase the risk of delayed diagnosis and more severe baseline disease. Furthermore, despite the widespread implementation of DOTS strategies, treatment supervision and follow-up intensity in remote rural areas may be compromised compared to urban centers due to resource constraints. Additionally, even with health insurance coverage, the financial burden of tuberculosis treatment disproportionately impacts low-income farming households. This can compromise nutritional support and treatment adherence, thereby increasing the risk of relapse. Clinically, patients with a bacteriologically positive diagnosis at the index episode faced a higher risk of recurrence. A positive baseline pathogen test indicates a higher initial bacterial load; if not sterilized completely, residual strains may serve as a source of recurrence (44). This aligns with findings that cultural positivity, particularly delayed conversion, predicts recurrence (45). Our study also identified protective factors related to healthcare access. Patients detected through active screening had a significantly lower recurrence risk compared to symptomatic visits, and those visiting municipal-level hospitals fared better than those at county-level institutions. These findings suggest that active case finding allows for diagnosis at earlier disease stages (46), and higher-level medical institutions may offer superior diagnostic and treatment quality, thereby reducing the likelihood of recurrence.

Although 83.1% of patients with recurrent PTB in our study achieved treatment success, this figure remains below the 85% target set by the Revised National Tuberculosis Control Programme (47), underscoring the importance of optimizing therapeutic strategies. Multivariable analysis identified advanced age (≥65 years) as an independent predictor of adverse outcomes, likely attributable to immunosenescence and comorbidities. In contrast, other studies (46), alongside HIV coinfection (48), consistently identify it as a critical driver of treatment failure. Beyond biological factors, treatment adherence is a pivotal determinant; poor compliance (23) and treatment interruption (26) significantly escalate reactivation risks. Additionally, adverse drug reactions (24) and the use of fixed-dose combinations may complicate therapeutic courses. Suboptimal outcomes in recurrence may also stem from a higher prevalence of acquired drug resistance (49) or pathological lung changes that impede drug penetration (50, 51). Consequently, we suggest that for high-risk patients, particularly the older adults, clinical management could be optimized by considering regimen intensification or extending the consolidation phase, coupled with enhanced post-treatment surveillance.

The demographic profile of tuberculosis in Quzhou has shifted significantly over the 18-year study period. We observed a consistent rise in the proportion of recurrent PTB cases among individuals aged ≥65 years, a trend that reflects the region’s aging population. The older population was identified as the main risk group for recurrence and poor re-treatment results in our model (Table 3). Apart from the overall risk level, our subgroup analysis also highlights the significant interaction between age and occupational risk. Among younger adults, farming is a strong predictor of recurrence; however, this association was not significant in the older adults, and there are significant interactions (Supplementary Figure 5). This observation shows that the influence of biological aging effectively outweighs the influence of socio-economic factors in later life. While the temporal pattern of early recurrence may suggest a potential role for endogenous reactivation due to waning immunity, we should interpret this with caution. Given the high TB burden in rural Western Zhejiang and the identified risk among farmers, exogenous reinfection still maybe a plausible mechanism driven by ongoing transmission and environmental exposure. The lack of molecular genotyping data in our study prevents us from definitively distinguishing between relapse (reactivation) and reinfection. Therefore, the observed recurrence patterns in our study likely reflect a complex interplay of biological vulnerability in the older adults and environmental exposure risks in rural settings. Future studies integrating genomic epidemiology are essential to quantify the specific contribution of each mechanism.

A critical finding of our analysis is the temporal robustness of the identified risk determinants. Despite the evolution of TB control policies over 18 years, the ‘study period’ was not an independent driver of recurrence or outcomes in our multivariable models, suggesting that biological determinants (e.g., immunosenescence) and clinical severity (e.g., bacterial load) override programmatic changes. However, our stratified analysis offered nuanced insights: the rising risk among farmers in recent years mirrors the concentration of the disease burden in rural areas, while the attenuated prognostic risk in the older adults during the second period may reflect improvements in geriatric care and supportive management.

In the multivariate model, study period (2007–2016 vs. 2017–2024) did not emerge as an independent predictor of recurrence or adverse outcomes. This observation coincided with a decline in overall reporting rates. Given that standard DOTS strategies effectively curbed transmission in the general population, the residual disease burden likely concentrated among clinically refractory core populations characterized by higher immune senescence and multiple comorbidities, thereby offsetting overall improvements in healthcare delivery. However, our stratified analysis revealed critical epidemiological shifts. Notably, the association between agricultural occupation and recurrence risk strengthened in recent years compared to earlier periods. This trend suggests disease burden is shifting toward rural areas, likely driven by widening disparities in healthcare access and socioeconomic resilience between urban and rural settings. Conversely, regarding prognosis, the risk of adverse outcomes associated with advanced age (≥65 years) has significantly decreased in recent years compared to earlier period. This narrowing of the prognostic gap suggests improvements in the healthcare environment for older patients and enhanced post-treatment monitoring. In summary, these data underscore that while intrinsic biological susceptibility (e.g., age, bacteriological characteristics) remains constant, extrinsic socioeconomic drivers are evolving. This necessitates dynamically adjusting public health resources to better serve rural older populations.

Our study had several limitations. First, this study was based mainly on a retrospective analysis of TB Information Management System case data rather than a prospective cohort study, which may underestimate the proportion of patients with recurrent PTB. Second, as this study was based on routine surveillance records, detailed information on several potential risk factors was not available. Specifically, variables such as smoking history, alcohol use, body mass index, and household economic status were not systematically collected in the database. Consequently, we could not adjust for these unmeasured factors in our multivariable models, and the observed associations may be subject to residual confounding. Future prospective studies with more granular clinical and socioeconomic data are needed to validate our findings. Third, a significant limitation of this study is the lack of systematic data on drug susceptibility testing (DST) and rapid molecular assays, particularly for the earlier years of the study period (2007–2015). Due to the high proportion of missing data, we could not determine the true prevalence of baseline drug resistance for the entire cohort or evaluate the specific impact of multidrug-resistant tuberculosis (MDR-TB) on recurrence risks (52–54). Therefore, the risk factors identified in our study should be interpreted with caution, as they may partially reflect unmeasured confounding by drug resistance status. Fourth, our findings were limited by geographic location, which makes them less appropriate for individuals who have recently migrated or who have migrated to different regions. Finally, it is unclear whether recurrence was endogenous rekindling or exogenous reinfection, as genotyping data were not available, and differentiating both mechanisms is important for optimizing post-treatment strategies.

Despite these limitations, our study has several strengths. First, few studies have conducted epidemiological studies of recurrent PTB, and we collected 18 years of surveillance data in areas with a high prevalence of TB. Second, we comprehensively identified independent risk factors for recurrence and evaluated adverse treatment outcomes. Crucially, we documented a distinct “acceleration phenomenon” in repeated episodes, characterized by a significant shortening of the recurrence-free interval. Although existing policies cannot completely block the occurrence of recurrent PTB, there is a need for more intensive and targeted clinical management of patients requiring retreatment.

## 5 Conclusion

This study indicates that recurrent tuberculosis remains a persistent public health challenge in Quzhou City. Our analysis identified advanced age (≥65 years) as a dominant predictor associated with both recurrence susceptibility and adverse retreatment outcomes, while male sex and farming occupation were also significant risk factors. Notably, nearly half of the recurrent cases occurred within the first 2 years after treatment completion, and the recurrence-free interval shortened significantly with repeated episodes. These findings suggest that current TB control strategies could be optimized by prioritizing age-specific management and extended post-treatment surveillance for high-risk groups. We suggest that integrated care models focusing on geriatric support warrant further consideration to address the dual burden of recurrence and poor prognosis in this vulnerable population.

## Data Availability

The raw data supporting the conclusions of this article will be made available by the authors, without undue reservation.
