# Supplementary material for: Epidemiological analysis and risk factors of recurrent pulmonary tuberculosis in Western Zhejiang Province, China (2007–2024)
Source: Front Public Health. 2026 Mar 4;14:1777279. doi: 10.3389/fpubh.2026.1777279 (PMC12996061; doi:10.3389/fpubh.2026.1777279)

**Supplementary Figure 1. Spatiotemporal Distribution of Recurrent Pulmonary Tuberculosis in Quzhou, China, 2007–2024.**

The figure shows a heatmap depicting the longitudinal evolution of disease burden across six administrative districts in Quzhou City over the 18-year study period. The analysis included all verified recurrent pulmonary tuberculosis (PTB) episodes (second episode or later). The top panel shows the annual number of recurrent cases recorded in each county or district. The bottom panel shows the corresponding annual notification rates (per 100,000 resident population). Notification rates were calculated using the year and district-specific resident population as the denominator. Color intensity reflects disease burden, and the exact values are shown in each cell. In the top panel, warmer colors (orange to purple) indicate higher case counts; in the bottom panel, brighter colors (yellow to green) indicate higher notification rates. Administrative units are ordered as presented in the figure (geographically or alphabetically).


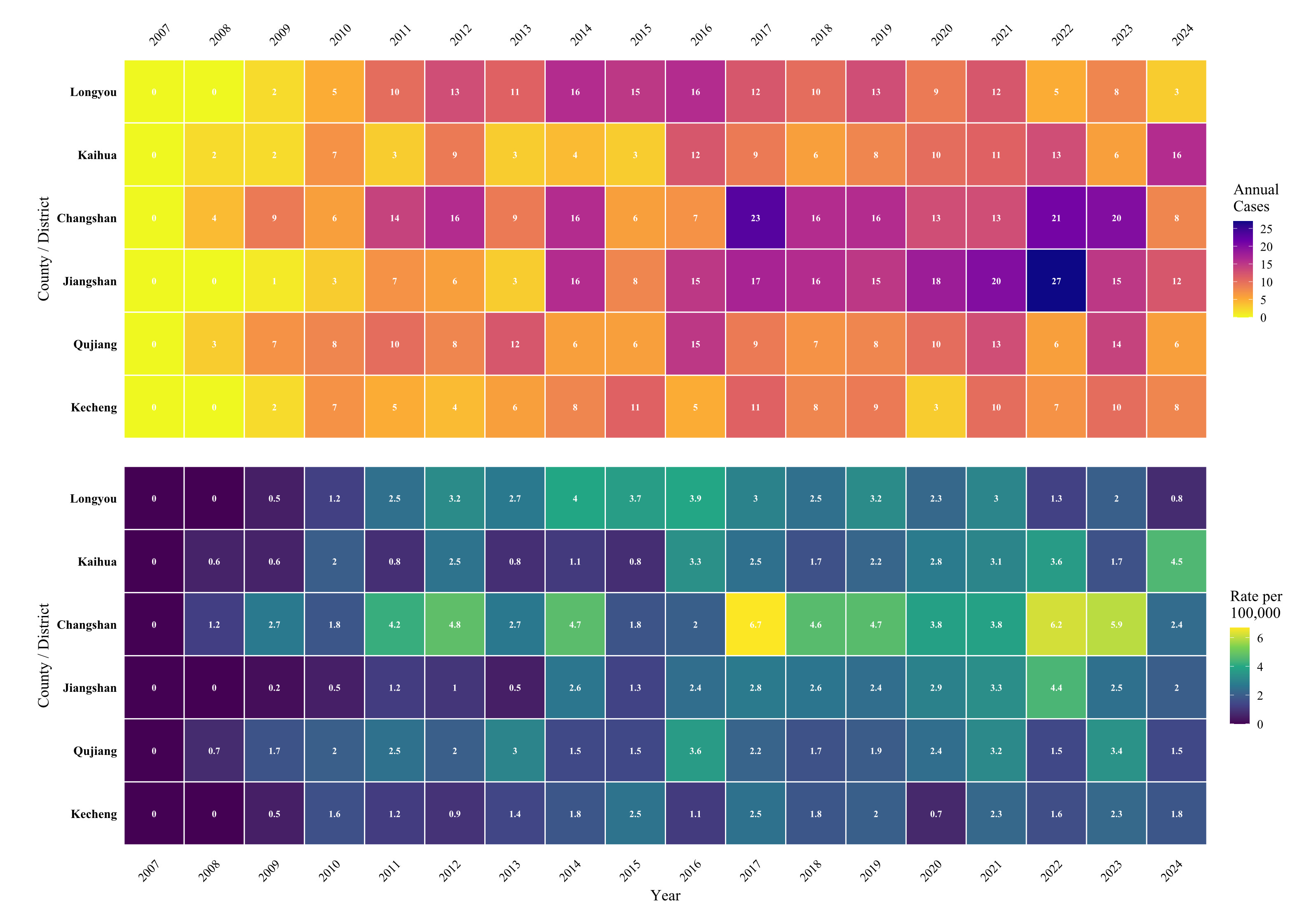


**Supplementary Figure 2. Temporal dynamics and seasonal periodicity of recurrent PTB in Quzhou, China.**

(A) Polar bar chart illustrating the monthly distribution of registered recurrent PTB cases. Radial bar length and color intensity (heatmap encoding) correspond to the absolute number of cases, with darker blue indicating higher counts. Numeric labels within the bars indicate the monthly case totals. The chart shows clear seasonality, with peaks in spring and summer. The number of episodes exceeds the number of patients (N=897) due to multiple recurrences in some individuals.

(B) Stacked bar chart summarizing the cumulative incidence by meteorological season. Seasonal variation was statistically significant (Chi-squared goodness-of-fit test: χ²=34.3, df=3, P < 0.001), indicating a non-uniform temporal distribution of recurrence.

**
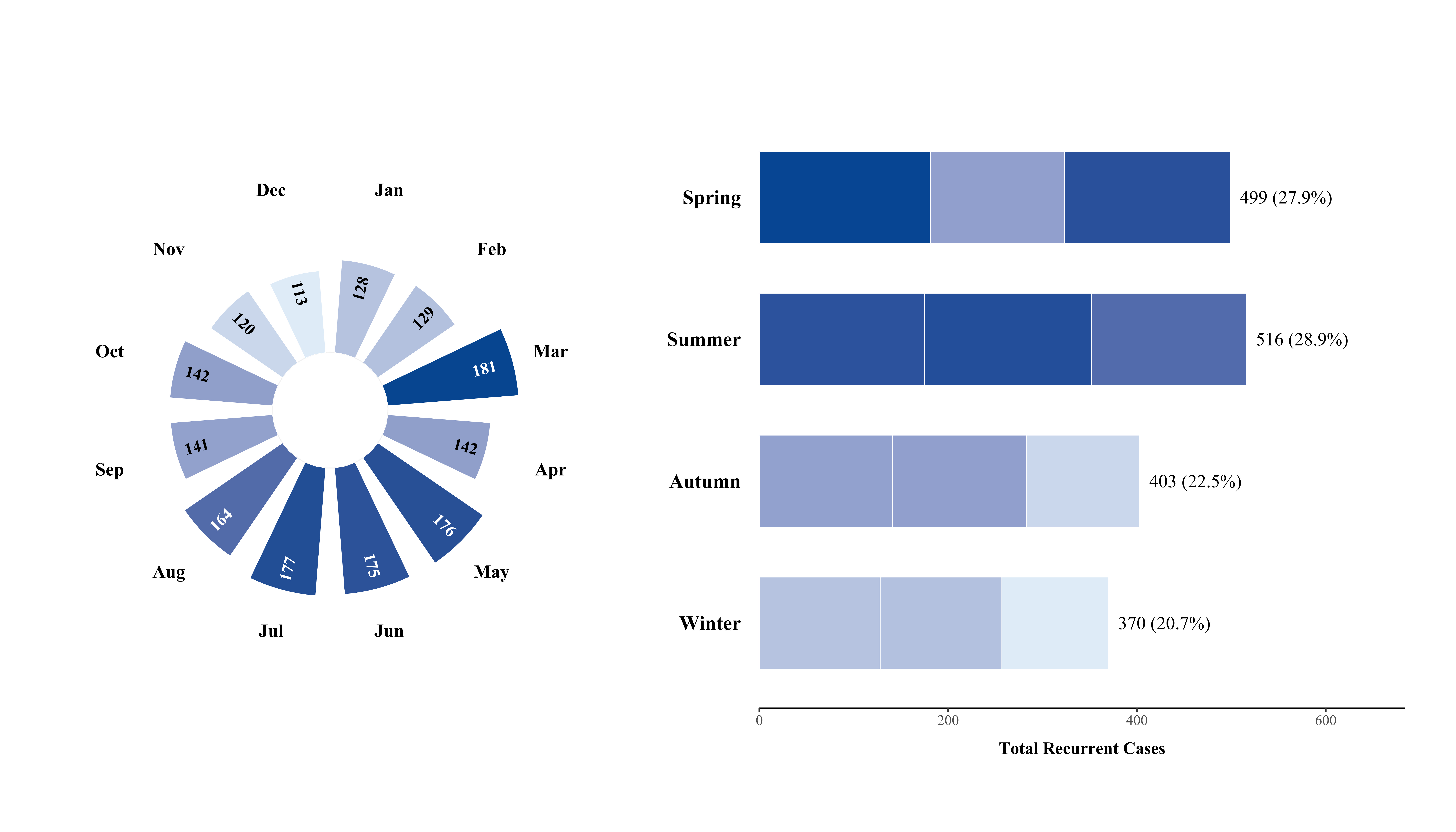
**

**Supplementary Figure 3. Univariate and Multivariable Analysis of Risk Factors for Tuberculosis Recurrence.**

Shown are the hazard ratios and 95% confidence intervals (CIs) for factors associated with the recurrence of tuberculosis. The left panel presents crude hazard ratios derived from univariate Cox proportional hazards models. The right panel displays adjusted hazard ratios from a multivariable model constructed using backward stepwise selection. Squares indicate point estimates, and horizontal bars indicate 95% CIs. The vertical dashed line represents a hazard ratio of 1.0; values to the right indicate an increased risk of recurrence, whereas values to the left indicate a protective effect. Em dashes in the multivariable analysis column indicate variables that were excluded from the final model. The x-axis is plotted on a logarithmic scale. This figure serves as a visual representation of the analysis presented in Table 2 of the main text. Please refer to Table 2 for the specific sample sizes (N) corresponding to each variable category.


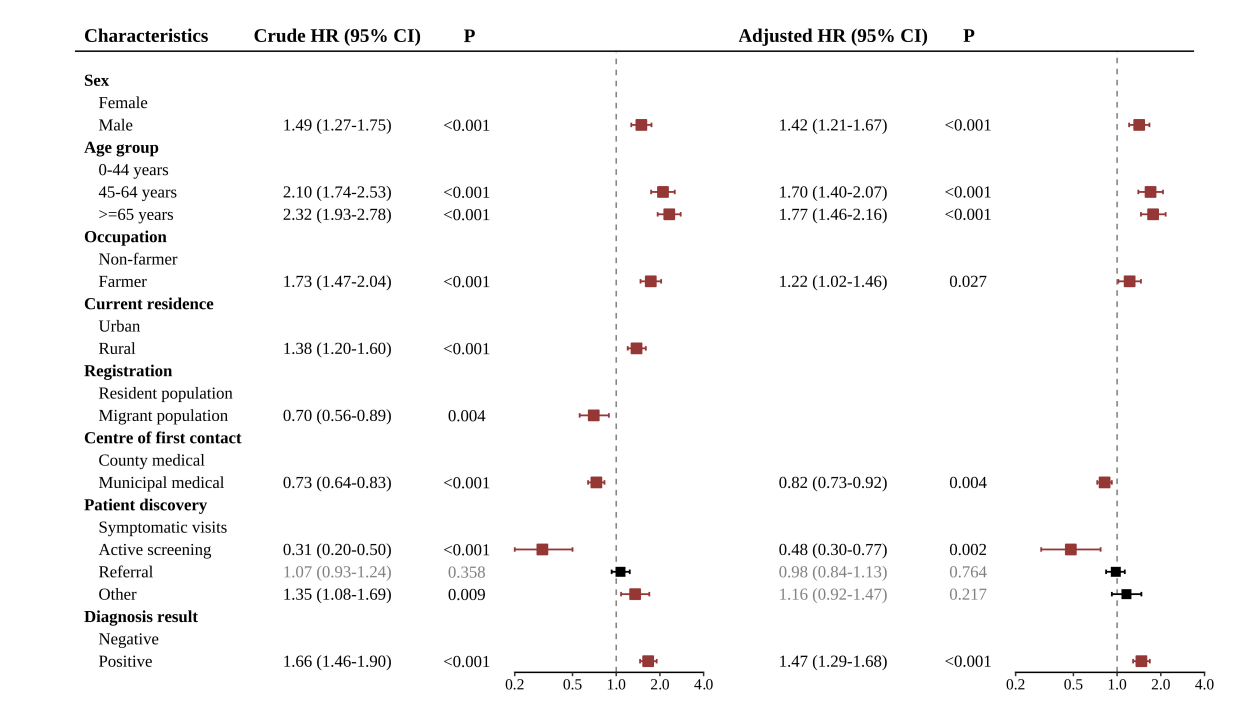


**Supplementary Figure 4. Geographic Distribution of Disease Burden and Recurrence Risk of Pulmonary Tuberculosis in Quzhou, China (2007–2024).**

(A) Choropleth map illustrating the cumulative disease burden. The color scale represents the absolute number of registered recurrent PTB cases in each of the six administrative districts. Darker orange/red hues indicate a higher total caseload. (B) Spatial heterogeneity of the recurrence notification rate (per 100,000 population). Rates were standardized using the 2016 registered resident population (total N=2,574,900) as the baseline denominator to account for demographic variations. The yellow-blue gradient depicts the intensity of recurrence risk, with darker blue districts exhibiting the highest rates. Administrative boundaries correspond to the standard geospatial data of Quzhou City.


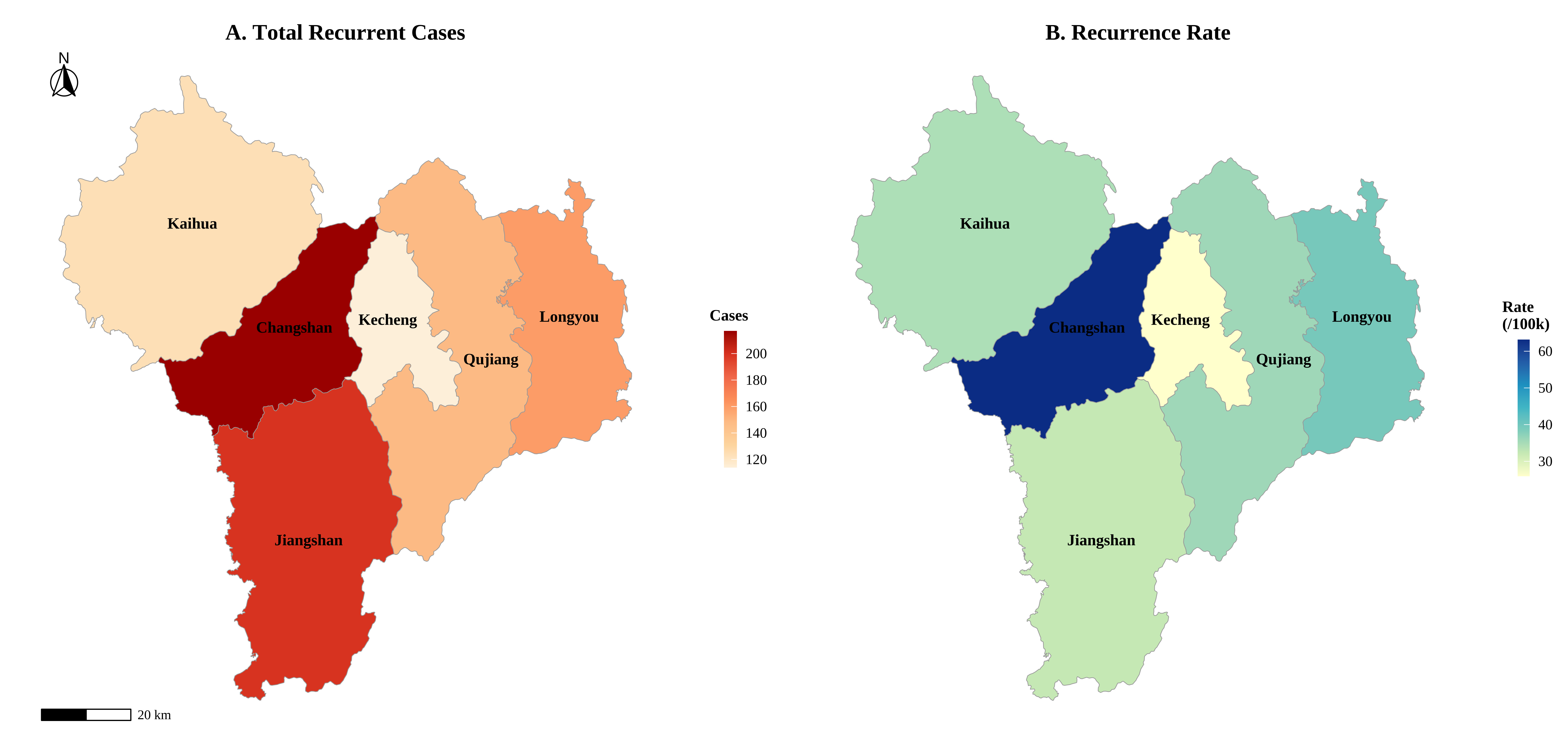


**Supplementary Figure 5. Subgroup Analyses of Key Risk Factors for Tuberculosis Recurrence in the Total Study Cohort.**

Data source and metrics: The analysis included the total cohort of 28,458 patients. Hazard ratios (HRs) and 95% confidence intervals (CIs) were derived from Cox proportional hazards models. Squares indicate point estimates; horizontal lines indicate 95% CIs. The vertical dashed line at 1.0 represents the null effect. Statistical interpretation: $P_{int}$ (P value for interaction) tests the heterogeneity of the risk factor's effect across different subgroups. $P_{int}>0.05$ indicates a consistent effect (robustness). $P_{int}<0.05$ (highlighted in red) indicates that the effect size differs significantly between subgroups (effect modification).


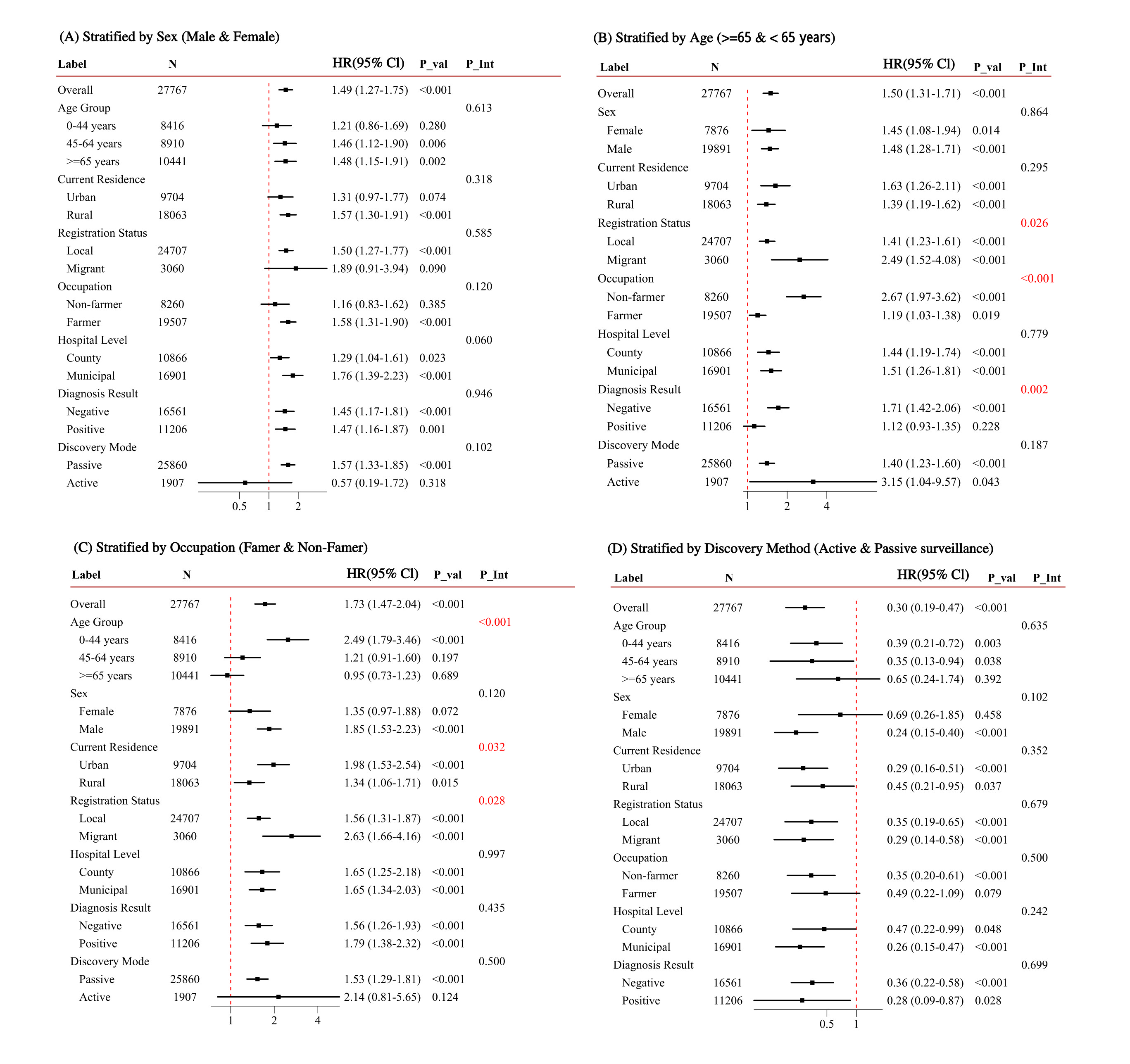


Panel-specific findings:

1. Age and Occupation Interactions (Panels B & C): Significant interactions were observed between age and occupation ($P_{int}<0.001$). The excess risk associated with farming was pronounced in younger adults (0-44 years, HR=2.49) but disappeared in the elderly ($\geq$65 years, HR=0.95), suggesting that biological aging and immunosenescence override socioeconomic drivers in later life (the "leveling effect"). Similarly, aging had a stronger impact on migrants (HR=2.49) compared to local residents (HR=1.41), indicating the loss of the "healthy migrant effect" in the elderly population.
2. Active Screening (Panel D): The protective effect of active screening (including health checkups) was robust and consistent across all subgroups ($P_{int}>0.05$), with HRs ranging from 0.28 to 0.47. Notably, the protection conferred by active screening was comparable between high-risk rural populations (HR=0.45) and urban populations (HR=0.29), supporting the prioritization of active case-finding in rural elderly communities.
3. Sex (Panel A): Male sex remained a significant risk factor across all subgroups ($P_{int}>0.05$, HR=1.49). The consistency of this risk, even among non-farmers working indoors, suggests underlying biological mechanisms (e.g., sex hormones) or deeply rooted behavioral factors (e.g., smoking) rather than environmental exposure alone.

**Supplementary Figure 6. Kernel Density Estimates of Recurrence-free Intervals according to Age Group.**

The figure shows the probability density curve of the no-relapse interval (in months) of 897 patients who were confirmed to have had their first recurrence. The analysis distinguishes three age groups: 0 to 44 years old (cyan), 45 to 64 years old (yellow) and 65 years old and above (red). The y-axis represents the probability density, estimated using a Gaussian kernel function. The height of the curve corresponds to the relative likelihood of recurrence occurring at a specific time point, such that the total area under each curve equals 1. The vertical dotted line represents the "Median Recurrence-free Interval" of each age group. All three age groups showed a very similar left-bias distribution, which was characterized by a sharp increase in the risk of recurrence and a significant peak between 12 and 24 months after the end of initial treatment.In this time cluster prediction of the previous two years, endogenous reactivation (usually earlier) rather than exogenous reinfection (random over time) is the main driver of recurrence throughout the life cycle.


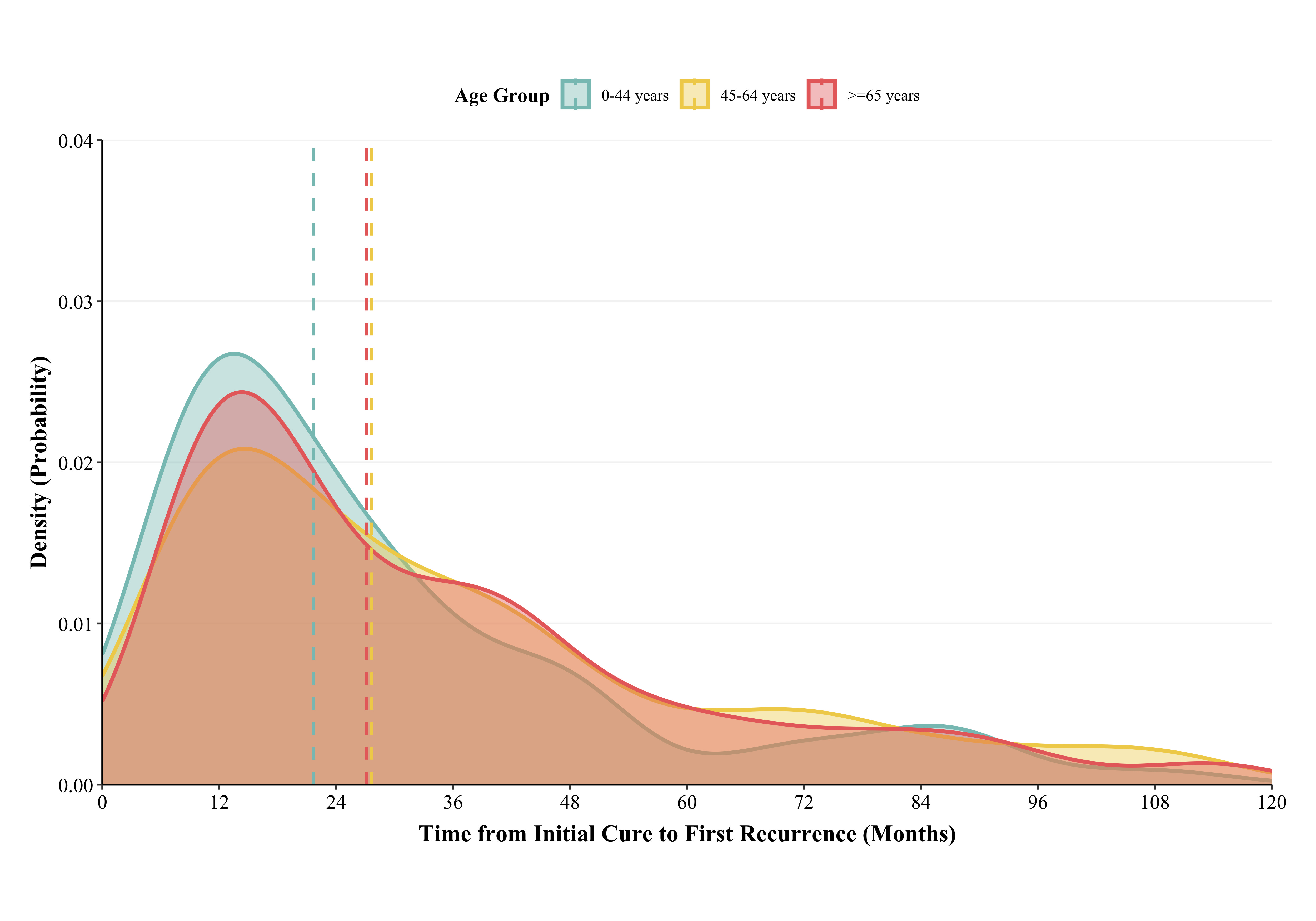


**Supplementary Figure 7. Kernel Density Estimates of Recurrence-free Intervals according to Sex Group.**

The figure shows the probability density curve of the no-relapse interval (in months) of 897 patients diagnosed with first recurrence. The analysis distinguishes between female patients (red) and male patients (blue). The y-axis represents the probability density and is estimated by the Gaussian nuclear function. The height of the curve corresponds to the relative possibility of recurrence at a specific point in time, so the total area under each curve is equal to 1. The vertical dotted line represents the "Median Recurrence-free Interval" of each gender. The distribution of men and women is highly consistent and there is a significant overlap. And the median interval between the two groups was almost the same, about 26.4 months. Similar to the analysis results in Supplementary Figure 5, the risk of both sexual recurrence increased significantly and peaked 12 to 24 months after the completion of initial treatment. Although this study found that men increase the risk of recurrence, it does not change the time dynamics of the disease recurrence, which suggests that there is a common early endogenous relapse biological mechanism between men and women.

**
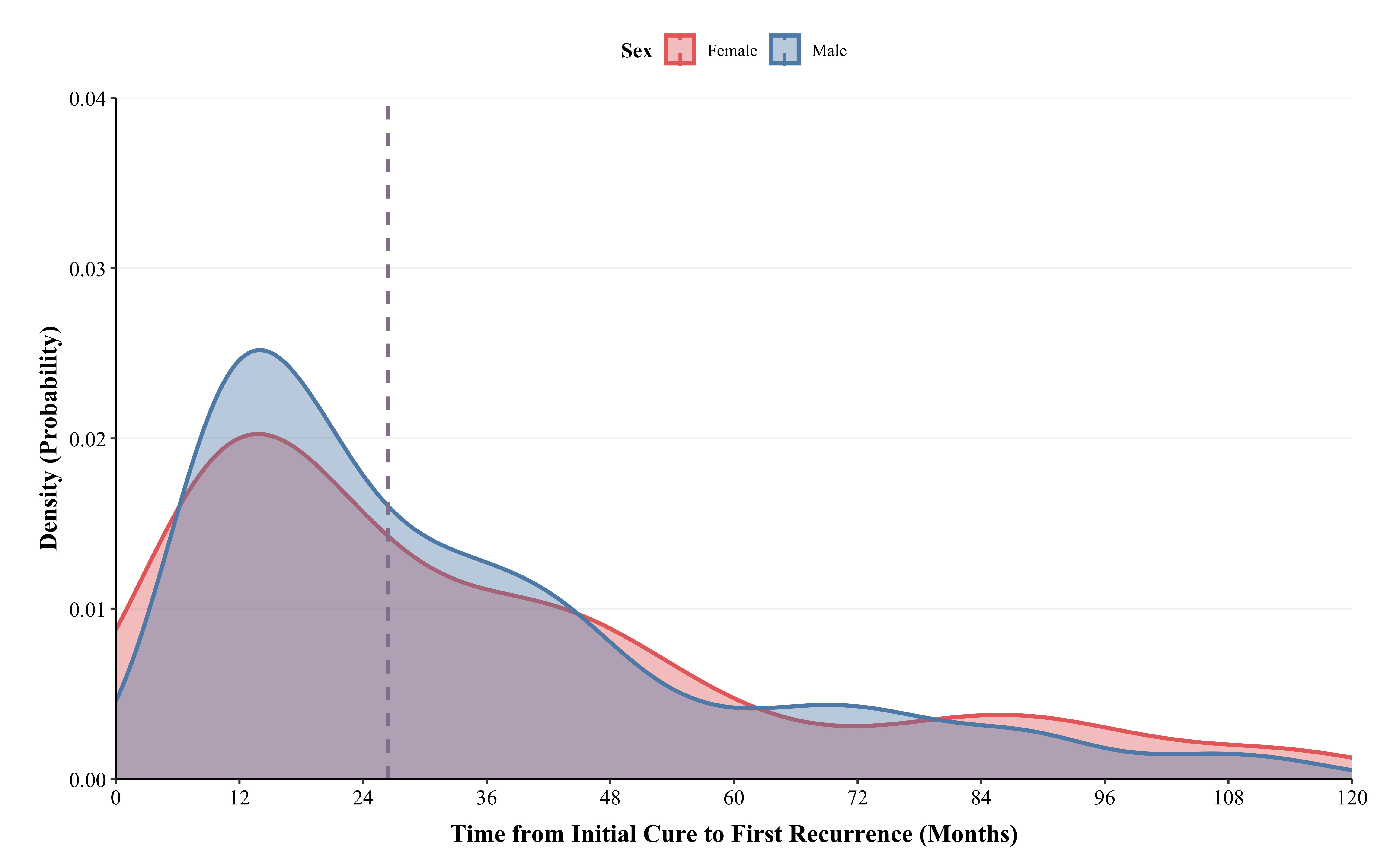
**

**Supplementary Figure 8. Temporal Evolution of Recurrence-Free Intervals among Patients with Recurrent PTB, 2007–2024.**

The figure shows the ridgeline density plots, which represents the time distribution of no recurrence interval (in months) of confirmed cases of recurrent PTB, and is stratified according to the interval of three years. The horizontal axis represents the time interval from the completion of the initial treatment to the diagnosis of recurrence. The height of each ridge line represents the relative density of cases in a given time interval. The vertical white dotted line indicates the median interval of no recurrence in each period of time.


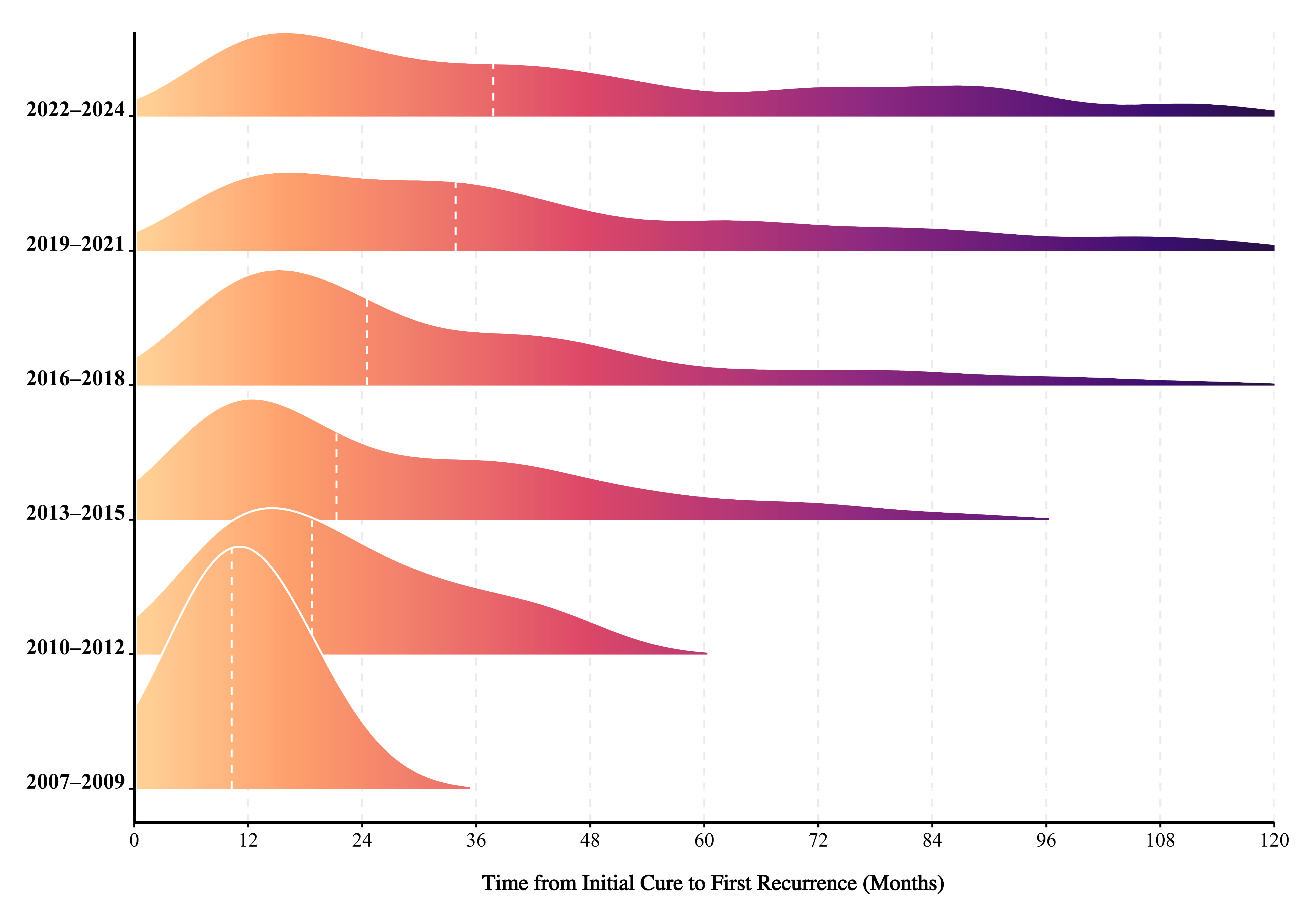


The data shows that the median no-relapse interval tends to shift to the right, from about 12 months in 2007-2009 to more than 36 months in 2022-2024. This time change trajectory shows that there are three potential mechanisms: The reduction of early recurrences reflects the improvement of the quality of management of patients with recurrent PTB in Quzhou City, effectively curbing early endogenous recurrence caused by incomplete sterilization. And the observed extension of the no-relapse interval is partly due to the maturity of the monitoring system, which makes it possible to capture late-stage recurrence events that cannot be observed in the early stage of the study. Surveillance System Maturation: The emergence of a long right-sided tail in later years is partially attributable to the maturation of the longitudinal database, enabling the capture of late-recurrence events (latency >10 years) that were unobservable during the early phases of the study.

With the extension of the interval (longer tail), the proportion of late recurrence increases. According to the epidemiological law, the possibility of exogenous reinfection after 2 years of stopping the drug gradually increases(1), which suggests that the focus of prevention and control is not only to prevent re-ignition (for example, for patients), but also to prevent re-infection (for example, to cut off community transmission) , protect the vulnerable elderly)

**Supplementary Table 1. Stratified Multivariable Cox Proportional Hazards Analysis by Study Period.**

**(A) Risk Factors for Tuberculosis Recurrence (Comparing Table 2)**

| **Characteristics** | **Original Model**  **Adjusted HR (95% CI)** | **Model Adjusted for Period**  **Adjusted HR (95% CI)** | **Change in HR** |
| --- | --- | --- | --- |
| **Study Period (2017–2024)** | Not included | 0.88 (0.75-1.04) | — |
| **Sex (Male)** | **1.42 (1.21-1.67)** | **1.41 (1.20-1.66)** | -0.7% |
| **Age Group** |  |  |  |
| **45–64 years** | **1.70 (1.40-2.07)** | **1.72 (1.41-2.10)** | +1.2% |
| $\text{≥}$**65 years** | **1.77 (1.46-2.16)** | **1.79 (1.48-2.18)** | +1.1% |
| **Occupation (Farmer)** | **1.22 (1.02-1.46)** | **1.21 (1.01-1.44)** | -0.8% |
| **First Contact (Municipal)** | **0.82 (0.73-0.92)** | **0.82 (0.72-0.95)** | 0.0% |
| **Discovery (Active screening)** | **0.46 (0.28-0.74)** | **0.49 (0.30-0.78)** | +6.5% |
| **Diagnosis (Positive)** | **1.38 (1.20-1.58)** | **1.49 (1.30-1.71)** | +8.0% |

**(B) Factors Associated with Adverse Treatment Outcomes (Comparing Table 3)**

| **Characteristics** | **Original Model**  **Adjusted HR (95% CI)** | **Model Adjusted for Period**  **Adjusted HR (95% CI)** | **Change in HR** |
| --- | --- | --- | --- |
| **Study Period (2017–2024)** | Not included | 0.78 (0.55-1.10) | — |
| **Sex (Male)** | **1.42 (1.21–1.67)** | **1.41 (1.20–1.66)** | -0.7% |
| **Age Group** |  |  |  |
| **45–64 years** | 1.01 (0.53–1.92) | 0.98 (0.51–1.88) | -3.0% |
| $\text{≥}$**65 years** | **2.06 (1.16–3.67)** | **1.91 (1.07–3.40)** | -7.3% |
| **Residence (Rural)** | — | 1.68 (0.95–2.96) | — |
| **Discovery (Active screening)** | **0.57 (0.38–0.84)** | **0.48 (0.30–0.77)** | -15.8% |
| **Diagnosis (Positive)** | **1.47 (1.29–1.68)** | **1.42 (1.00–2.01)** | -3.4% |

This analysis was conducted to address potential temporal heterogeneity in tuberculosis control practices over the 18-year study duration. The primary multivariable model presented in the main text (Table 2 and Table 3), constructed using backward stepwise selection based on AIC. The same model fitting process but with the mandatory inclusion of "Study Period" (2007-2016 vs. 2017-2024) as a candidate covariate in the initial step.

In both recurrence and outcome models, the "Study Period" variable was not statistically significant (P > 0.05) and was subsequently excluded during the stepwise selection process. Dashes (—) indicate variables that were not retained in the final parsimonious model. Note that "Rural Residence" was retained in the period-adjusted outcome model (Panel B) with borderline significance, whereas it was excluded in the original model; however, the direction of effect (risk factor) remained consistent.

The adjustment for study period did not materially alter the magnitude (HRs) or statistical significance of the core predictors, confirming the robustness of the main findings. Bold text indicates statistical significance (P<0.05). Abbreviations: HR, hazard ratio; CI, confidence interval.

**Supplementary Table 2. Comparison of Independent Risk Factors across Different Study Periods.**

**A. Risk Factors for Tuberculosis Recurrence (Comparing Table 2 Results)**

| **Characteristics** | **Total Cohort (2007-2024)**  **(N=28,458)** | **Period 1 (2007-2016)**  **(N=16,913)** | **Period 2 (2017-2024)**  **(N=11,545)** |
| --- | --- | --- | --- |
| **Sex** |  |  |  |
| Female | Reference | Reference | Reference |
| Male | **1.42 (1.21-1.67)** | **1.39 (1.15-1.67)** | **1.49 (1.06-2.07)** |
| **Age** |  |  |  |
| 0-44 years old | Reference | Reference | Reference |
| 45-64 years old | **1.70 (1.40-2.07)** | **1.74 (1.41-2.16)** | **2.07 (1.27-3.37)** |
| >=65 years old | **1.77 (1.46-2.16)** | **1.95 (1.58-2.41)** | **1.74 (1.07-2.82)** |
| **Occupation** |  |  |  |
| Non-farmer | Reference | Reference | Reference |
| Farmer | **1.22 (1.02-1.46)** | — | **1.66 (1.12-2.45)** |
| **Centre of first contact** |  |  |  |
| County medical institutions | Reference | Reference | Reference |
| Municipal medical institutions | **0.82 (0.73-0.92)** | **0.81 (0.69-0.96)** | 0.77 (0.58-1.02) |
| **Patient discovery method** |  |  |  |
| Symptomatic visits | Reference | Reference | Reference |
| Active screening | **0.48 (0.30-0.77)** | **0.34 (0.19-0.61)** | — |
| **Diagnosis** |  |  |  |
| Negative | Reference | Reference | Reference |
| Positive | **1.47 (1.29-1.68)** | **1.44 (1.23-1.67)** | **1.73 (1.30-2.31)** |

**B. Risk Factors for Adverse Treatment Outcomes (Comparing Table 3 Results)**

| **Characteristics** | **Total Cohort (2007-2024)**  **(N=1,348)** | **Period 1 (2007-2016)**  **(N=897)** | **Period 2 (2017-2024)**  **(N=451)** |
| --- | --- | --- | --- |
| **Age** |  |  |  |
| 0-44 years old | Reference | Reference | Reference |
| 45-64 years old | 1.01 (0.53-1.92) | 1.22 (0.47-3.16) | 0.77 (0.32-1.87) |
| >=65 years old | **1.92 (1.15-3.19)** | **2.51 (1.07-5.90)** | 1.55 (0.71-3.41) |
| **Current residence** |  |  |  |
| Urban | Reference | Reference | Reference |
| Rural | — | — | 1.68 (0.95-2.96) |
| **Patient discovery method** |  |  |  |
| Symptomatic visits | Reference | Reference | Reference |
| Active screening | **0.57 (0.38-0.84)** | — | — |
| **Diagnosis** |  |  |  |
| Negative | Reference | Reference | Reference |
| Positive | **1.66 (1.18-2.34)** | **2.63 (1.56-4.44)** | **1.42 (1.00-2.01)** |

Data are presented as Adjusted Hazard Ratios (aHR) with 95% Confidence Intervals. Bold text indicates statistical significance (*P* < 0.05). Multivariable Cox proportional hazards models were constructed using a backward stepwise selection procedure based on the Akaike Information Criterion (AIC). Em dashes (—) indicate variables that were not selected in the final parsimonious model for that specific period or variables with insufficient events for convergence. "Active screening" includes both health checkups and active screening programs. "Positive" diagnosis refers to bacteriologically confirmed cases. Abbreviations: HR, hazard ratio; CI, confidence interval.

**Supplementary Table 3. Sensitivity Analysis of Multivariable Cox Proportional Hazards Models Adjusted for Study Period.**

Data are presented as adjusted hazard ratios (aHRs) with 95% confidence intervals (CIs). The analysis was conducted to assess the temporal robustness of the primary findings by forcing the "Study Period" variable (Period 1: 2007–2016 vs. Period 2: 2017–2024) into the multivariable models.

|  | **Recurrence Risk (N=28,458)** | | **Adverse Outcomes (N=891)** | |
| --- | --- | --- | --- | --- |
| **Characteristic**^1^ | **aHR (95% CI)**^1^ | **p-value**^1^ | **aHR (95% CI)**^1^ | **p-value**^1^ |
| Sex |  |  |  |  |
| Female | — |  | — |  |
| Male | 1.53 (1.30 to 1.80) | **<0.001** | 1.16 (0.76 to 1.76) | 0.493 |
| Age |  |  |  |  |
| 0-44 years | — |  | — |  |
| 45-64 years | 1.33 (1.09 to 1.61) | **0.005** | 0.98 (0.51 to 1.89) | 0.960 |
| >=65 years | 1.11 (0.91 to 1.35) | 0.314 | 1.99 (1.10 to 3.58) | **0.022** |
| Residence |  |  |  |  |
| Urban | — |  | — |  |
| Rural | 1.49 (1.28 to 1.74) | **<0.001** | 1.18 (0.79 to 1.75) | 0.411 |
| Registration |  |  |  |  |
| Local | — |  | — |  |
| Migrant | 0.65 (0.51 to 0.83) | **<0.001** | 0.92 (0.49 to 1.74) | 0.803 |
| Occupation Group |  |  |  |  |
| Non-farmer | — |  | — |  |
| Farmer | 1.31 (1.09 to 1.57) | **0.004** | 0.88 (0.57 to 1.36) | 0.573 |
| Hospital Level |  |  |  |  |
| County | — |  | — |  |
| Municipal | 0.69 (0.60 to 0.80) | **<0.001** | 1.24 (0.87 to 1.75) | 0.232 |
| Discovery Method |  |  |  |  |
| Passive | — |  | — |  |
| Active | 0.46 (0.29 to 0.73) | **<0.001** | 0.36 (0.09 to 1.45) | 0.150 |
| Diagnosis_Res |  |  |  |  |
| Negative | — |  | — |  |
| Positive | 1.02 (0.89 to 1.17) | 0.763 | 1.53 (1.07 to 2.19) | **0.021** |
| Study Period |  |  |  |  |
| Period1 (2007-2016) | — |  | — |  |
| Period2 (2017-2024) | — | 0.972 | 0.77 (0.55 to 1.08) | 0.132 |
| ^1^Models were adjusted for Study Period (2007-2016 vs 2017-2024). | | | | |
| Abbreviations: CI = Confidence Interval, HR = Hazard Ratio, aHR: Adjusted Hazard Ratio. | | | | |

**Supplementary Figure 9. Assessment of Proportional Hazards Assumption for Multivariable Cox Regression Models using Schoenfeld Residuals.**

The proportional hazards assumption was verified using the scaled Schoenfeld residuals test (cox.zph) and visual inspection of the plots. The x-axis represents the transformed time, and the y-axis represents the scaled Schoenfeld residuals (Beta(t)) for each covariate. The solid line represents a smoothing spline fit to the residuals, and the dashed lines represent the ±2 standard error bands. A horizontal trend in the spline fit indicates that the proportional hazards assumption holds.

(A) Schoenfeld residual plots for the multivariable model identifying risk factors for PTB recurrence (corresponding to Table 2 in the main text). The Global Schoenfeld Test result was P = 0.1095, indicating no violation of the assumption.

(B) Schoenfeld residual plots for the multivariable model identifying predictors of adverse retreatment outcomes (corresponding to Table 3 in the main text). The Global Schoenfeld Test result was P = 0.5684, indicating no violation of the assumption. Both models satisfied the proportional hazards assumption (Global P > 0.05).

**Supplementary Figure 9(A)**


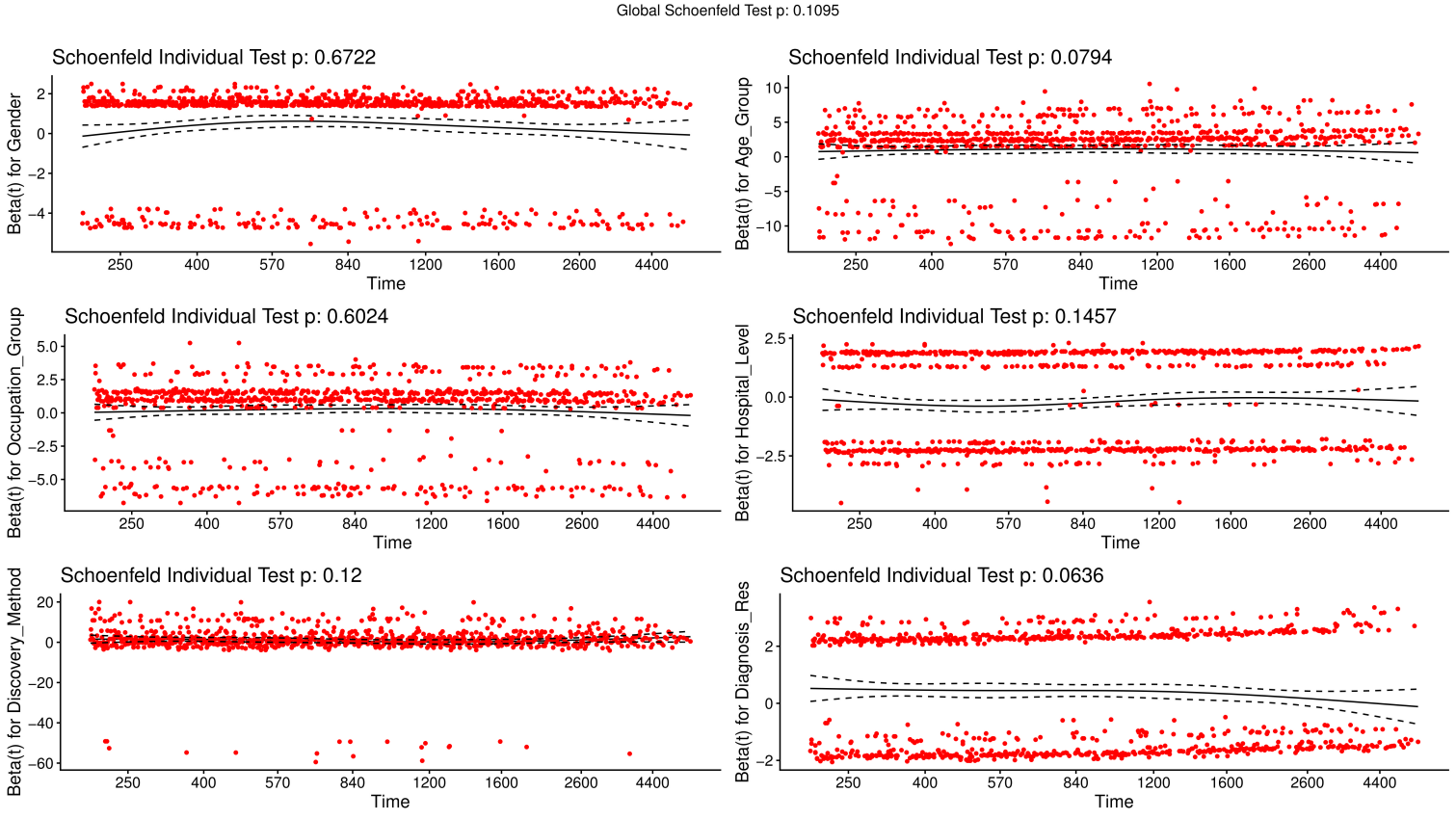


**Supplementary Figure 9(B)**


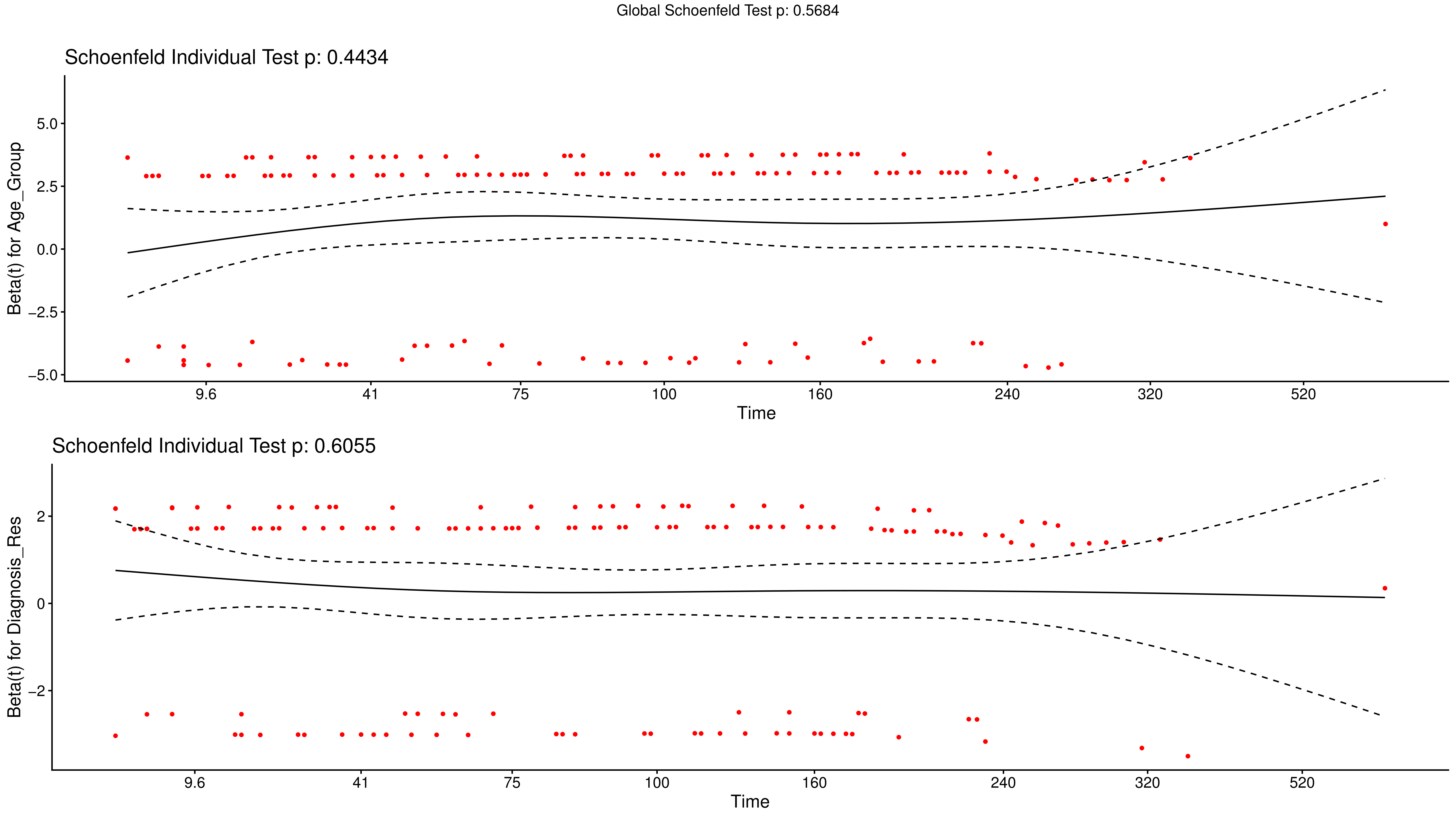

Supplement: Supplementary file 1 [file Data_Sheet_1.docx]
